# First genome-wide data from Italian European beech (*Fagus sylvatica* L.): Strong and ancient differentiation between Alps and Apennines

**DOI:** 10.1371/journal.pone.0288986

**Published:** 2023-07-20

**Authors:** Alexis Marchesini, Andrea Silverj, Sara Torre, Omar Rota-Stabelli, Matteo Girardi, Iacopo Passeri, Ilaria Fracasso, Federico Sebastiani, Cristiano Vernesi

**Affiliations:** 1 Institute for Sustainable Plant Protection (IPSP), The National Research Council of Italy (CNR), Sesto Fiorentino (Florence), Italy; 2 Research Institute on Terrestrial Ecosystems (IRET), The National Research Council of Italy (CNR), Porano (Terni), Italy; 3 NBFC, National Biodiversity Future Center, Palermo, Italy; 4 Centre Agriculture Food Environment, University of Trento, San Michele all’Adige, Italy; 5 Department CIBIO, University of Trento, Trento, Italy; 6 Plant Protection Unit, Research and Innovation Centre, Fondazione Edmund Mach, S. Michele all’Adige (Trento), Italy; 7 Conservation Genomics Unit, Research and Innovation Centre- Fondazione Edmund Mach, S. Michele all’Adige (Trento), Italy; 8 Forest Ecology Unit, Research and Innovation Centre- Fondazione Edmund Mach, S. Michele all’Adige (Trento), Italy; 9 Faculty of Science and Technology, Free University of Bolzano-Bozen, Bolzano, Italy; Central University of Kerala, INDIA

## Abstract

The European beech (*Fagus sylvatica* L.) is one of the most widespread forest trees in Europe whose distribution and intraspecific diversity has been largely shaped by repeated glacial cycles. Previous studies, mainly based on palaeobotanical evidence and a limited set of chloroplast and nuclear genetic markers, highlighted a complex phylogeographic scenario, with southern and western Europe characterized by a rather heterogeneous genetic structure, as a result of recolonization from different glacial refugia. Despite its ecological and economic importance, the genome of this broad-leaved tree has only recently been assembled, and its intra-species genomic diversity is still largely unexplored. Here, we performed whole-genome resequencing of nine Italian beech individuals sampled from two stands located in the Alpine and Apennine mountain ranges. We investigated patterns of genetic diversity at chloroplast, mitochondrial and nuclear genomes and we used chloroplast genomes to reconstruct a temporally-resolved phylogeny. Results allowed us to test European beech differentiation on a whole-genome level and to accurately date their divergence time. Our results showed comparable, relatively high levels of genomic diversity in the two populations and highlighted a clear differentiation at chloroplast, mitochondrial and nuclear genomes. The molecular clock analysis indicated an ancient split between the Alpine and Apennine populations, occurred between the Günz and the Riss glaciations (approximately 660 kyrs ago), suggesting a long history of separation for the two gene pools. This information has important conservation implications in the context of adaptation to ongoing climate changes.

## 1. Introduction

The European beech (*Fagus sylvatica* L.) is one of the most common and widely distributed broadleaved tree species in Europe. It highly contributes to European forest biodiversity, acting as a foundation and keystone species and providing many ecosystem services [1]. In its ample natural range, the European beech experiences a wide range of ecological conditions, showing evidence of adaptations to climate at the regional scale [2] and for altitudinal gradients [3]. Despite its adaptability, the species is sensitive to both severe drought and flooding and exhibits strong trait-environment interactions: it is therefore expected to be particularly vulnerable to the effects of global warming [4, 5]. Indeed, growth declines driven by climate change have already been documented in recent decades across a large portion of its distribution, particularly in southern Europe [6].

The broad ecological niche of this tree species reflects a complex past evolutionary history, shaped by the multiple glacial-interglacial cycles. Specifically, the integration of palaeobotanical and genetic data revealed the long-term survival of the European beech in different Quaternary glacial refugia and different routes of postglacial spread, where the Mediterranean refuge areas (Southern Balkan, Italian and Iberian Peninsula) did not contribute to the recolonization of central and northern Europe [7], although more recent studies highlighted a contact zone among different lineages in this area, indicating a potential additional refugium located in Central-Eastern Europe [8–10]. In the Italian peninsula, the complex orography of Alps and Apennines gave rise to local microrefugia [11]: as a consequence, Italian populations display high genetic diversity with a strong geographical structure. In fact, a clear differentiation was shown between northern Italian populations along the Alps and central-southern Italian populations [12]. Although several studies have already been carried out, until recently, inferences on beech genetic variability and population structure were limited by the number of markers used: genetic data were based on polymorphisms of few chloroplast [7, 12, 13] and neutral nuclear markers [7, 14, 15]; see [10] for a recent meta-analysis on microsatellite data. Only in recent times the discovery of single nucleotide polymorphisms (SNPs) in coding regions has allowed the investigation of adaptation to specific environmental conditions [2, 3, 8, 16–19]. However, these studies have been conducted on a limited set of polymorphisms within selected genes; moreover, the geographic coverage does not embrace the whole distributional range of the species, being concentrated in the French Alps and Switzerland. Genomic information from the marginal part of the species distribution is therefore still lacking, although these geographic areas might be of particular importance for understanding the legacy of evolutionary history on the genetic diversity of the European beech [10] and assessing its adaptation potential to climate change [20–22].

Following the high-throughput sequencing revolution, a large number of genome sequencing projects have been completed in the last two decades, covering a wide variety of model and non-model species [23]. In the wake of this, in 2018 the first assembly of *Fagus sylvatica* genome sequence has been released [24], followed in 2022 by a chromosome level assembly [25]. Newly available reference genomes allow the whole-genome genotyping of individual samples, paving the way to genome-wide population genomic approaches. Whole genome scanning at population scale can reveal loci that control adaptive differences among natural populations [26] with high precision. On the one hand, this information can be used to develop more effective management and conservation strategies, aimed at reducing the impact of climate change and other stressors (e.g., newly emerging diseases); on the other hand insights of the genetic mechanisms linked to local adaptation can be obtained [27]. Moreover, the growing availability of complete genomes, including chloroplast and mitochondrial ones, from different species and locations provides effective resources for phylogenetic and phylogeographic studies.

In the European beech, the role of glaciations and different refugia in shaping the current distribution of genetic diversity is not yet fully elucidated [7]: more accurate phylogenetic and molecular clock inferences, based on entire genomes, could be used to confidently date the split between different evolutionary lineages and to more accurately interpret phylogeographic patterns [28]. In this study, we conducted whole genome resequencing of nine *F*. *sylvatica* samples from two Italian locations in the Alpine and Apennine mountain ranges, respectively, with the aims of: (1) assessing the current levels of genome-wide diversity for the species in the Italian peninsula, at both organellar and nuclear genomes (including the investigation of functional diversity patterns); (2) testing the previously identified distinctiveness [7, 8] of Alpine and Apennine beech populations; and (3) accurately estimating the time of their evolutionary split.

## 2. Materials and methods

### 2.1. Sample selection, DNA isolation and sequencing

**Fig 1:**
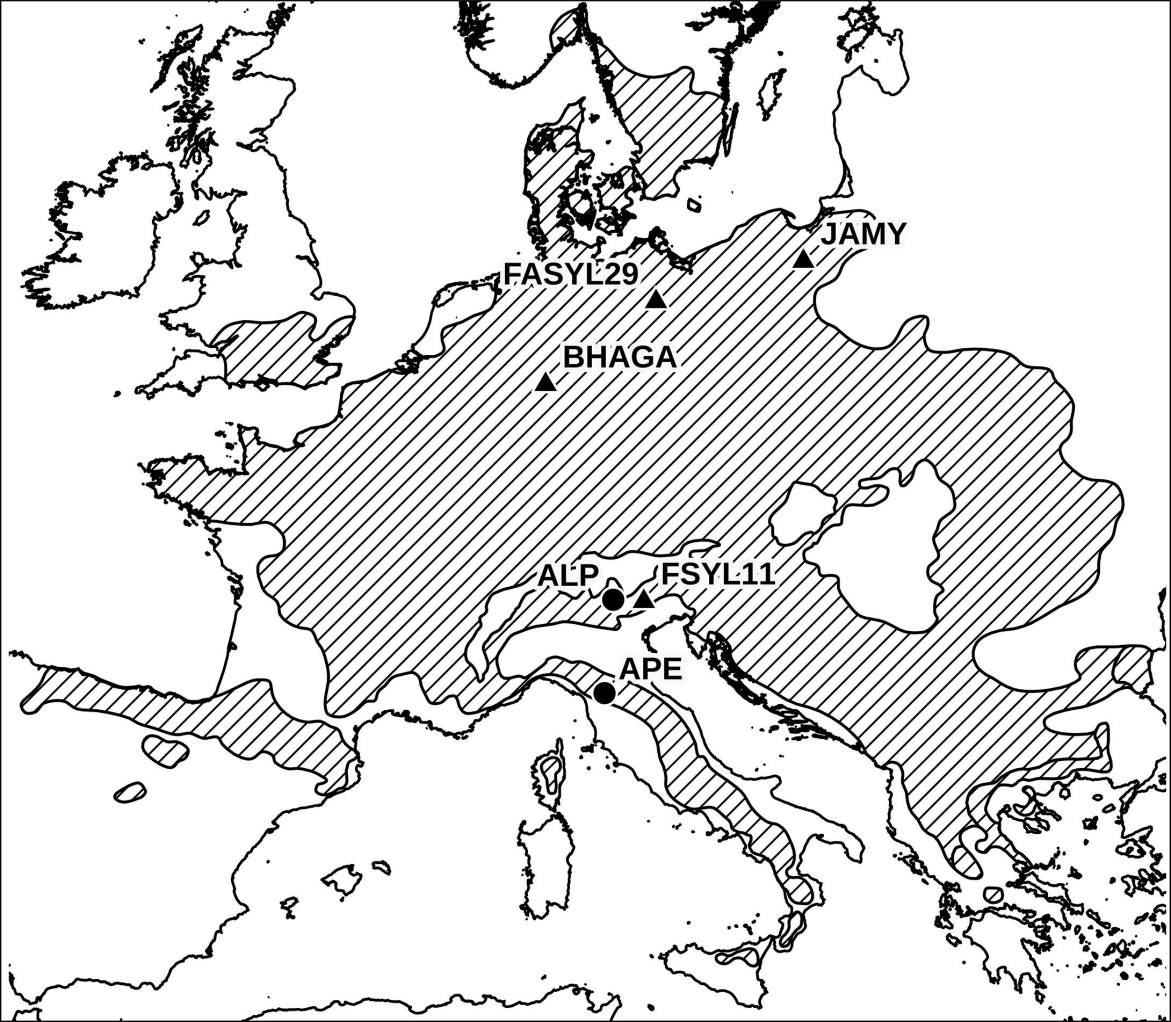
Map of the European beech samples included in the study. Circles represent the Italian stands sampled in the present studies (ALP, N = 4 and APE, N = 5); triangles represent individuals with publicly available nuclear (BHAGA and JAMY) and organellar (BHAGA, JAMY, FSYL11 and FASYL29) genomes, which were included in some analyses (see section 2.2); the map was generated using QGIS v3.20 (http://www.qgis.org) and the hatched area represents the present-day distribution of *Fagus sylvatica* [29].

In the present study, nine individual trees from two distinct European beech stands located in the Italian peninsula (Alps and Apennines, respectively) were sampled (Fig 1 and S1 Table). Specifically, we collected fresh young leaf samples from Val di Cembra, in the south-eastern Alps (Lat 46.201753°, Long 11.209742°; hereafter: ALP; N = 4), and from Maresca (Foresta del Teso), in the northern Apennines (Lat 44.064725°, Long 10.857770°; hereafter: APE; N = 5). Both the sampled sites are native and naturally regenerated forests with no record of plantings using seeds of foreign origin, where *Fagus sylvatica* is the dominant or co-dominant tree species. In order to avoid the sampling of closely related individuals, we chose dominant adult trees growing at least 100m apart from each other; as a further check, pairwise relatedness coefficients (r) among individuals of the same stand were computed (see section 2.4.3). All conducted experiments complied with the current laws of Italy. No permission was required for sampling at the sites studied. Total genomic DNA was extracted from the leaves of each individual using the DNeasy Plant Mini Kit (QIAGEN), following the manufacturer’s protocol. Short-read Illumina whole-genome resequencing was performed at Novogene Inc., using an Illumina NovaSeq platform (PE150). The genomic DNA of each sample was randomly sheared into short fragments of about 350 bp, which were subjected to library construction using the NEBNext® DNA Library Prep Kit. The final libraries were purified, and library quality and size verification were assessed on an Agilent 2100 Bioanalyser (Agilent Technologies).

In addition, available reads derived from one previously sequenced German individual from the Kellerwald-Edersee National Park [24] (GenBank accession number: PRJEB24056; hereafter: BHAGA) and one Polish individual from the Jamy Nature Reserve [25] (GenBank accession number: PRJNA450822; hereafter: JAMY) were included in the dataset, for comparison.

### 2.2. Chloroplast and mitochondrial genome analysis

Organellar sequencing reads of the nine Italian beech samples were mapped to the chloroplast [30] and mitochondrial [31] reference genomes. The assembly of the chloroplast and mitochondrial genomes of each of the nine samples was performed with NovoPlasty [32]. In addition, the software Snippy v. 3.2-dev [33] was used for reference-based mapping, consensus generation and variant detection of all the samples. Chloroplast and mitochondrial genomes of the Italian samples sequenced in the present study were then aligned using MAFFT v.7 [34], together with the following reference samples: two German samples (BHAGA; [24] and FASYL29 [30, 31]) and one from Poland (JAMY; [35]). For the cpDNA dataset, one additional Italian sample from the eastern Alps (Veneto) whose chloroplast genome was sequenced in a previous study [28] (FSYL11; NCBI accession number: MW566783) was also included (for the geographical location of all reference samples, see Fig 1).

The resulting alignment was revised manually and haplotypes were defined as combinations of SNP and indel variants across the chloroplast/mitochondrial genomes. The R package *pegas* [36] was used for (i) haplotype estimates, (ii) haplotype diversity indices, and (iii) to construct minimum spanning networks of haplotypes (function “msn”).

### 2.3. Phylogenetic inference

#### 2.3.1. Data collection and dataset preparation for chloroplast and mitochondrial phylogenetic analysis

We collected all available chloroplast and mitochondrial genomes and proteomes for the genus *Fagus*, and a pool of chloroplast sequences of the genus *Castanea*, *Castanopsis* and *Quercus*. For mitochondrial data, we were unable to include more than one genus (*Quercus*) as an outgroup, given that only a few mitochondrial genomes were available. A list of all sequences downloaded from NCBI can be found in S2 Table.

For chloroplast data, one downloaded genome per species was used to prepare a dataset for a first-step molecular clock analysis. A second dataset comprising all *Fagus sylvatica* samples sequenced in this study (i.e., four samples from the Alps and five samples from the Apennines), the reference German and Italian samples (FASYL29 and FSYL11; NCBI reference numbers NC_041437.1 and MW566783.1, respectively; [37]) and *Fagus crenata* (NC_041252.1; [38]), identified as sister species in the first clock analysis, was prepared for a molecular clock calibrated using the topology and the estimates of the ages of the splits between different species obtained in the first analysis.

For mitochondrial data, given the scarce availability of genomes and calibrations that prevents from conducting a reliable phylogenetic study, only a dataset comprising the newly sequenced genomes was created. All genomes were quality checked using a custom Python script (https://github.com/andrea-silverj/BioinfoToolkit/blob/main/clean_seqs.py), removing low quality regions and unconventional bases.

#### 2.3.2. Orthology analysis

For the first chloroplast dataset, including the genomes of different Fagaceae genera (see section 2.3.1), we performed an orthology analysis with OrthoFinder v2.5.4 [39], identifying 80 orthogroups, with 57 single copy orthologs that were stored in separate fasta files as amino acid sequences.

#### 2.3.3. Sequence alignment and trimming

Amino acid sequences of chloroplast single copy orthologs were individually aligned using MAFFT v.7, with the “—auto” option. Aligned files were concatenated independently using FASconCAT [40], creating a final matrix for phylogenetic analysis.

For the second chloroplast dataset, including only *F*. *sylvatica* genomes and one *F*. *crenata* genome (identified as sister species; see section 2.3.1), sequence alignment was performed using the same method described above, followed by trimming with trimAl v1.2 [41] with the “-automated1” command, to remove non conserved and poorly aligned regions. Eventually, the mitochondrial dataset was aligned with the same strategy, but trimming was not necessary in this case, as genomes were very similar, and no misalignments were detected by a manual inspection.

#### 2.3.4. Maximum-likelihood phylogeny

Maximum-likelihood phylogenies were obtained for all matrices using RAxML [42] with options “raxmlHPC-PTHREADS-SSE3 -p 1989 -m PROTGAMMAAUTO -x 2483 -# 100 -f a -T 20” for amino acid data and “raxmlHPC-PTHREADS-SSE3 -p 1989 -m GTRCAT -x 2483 -# 100 -f a -T 16” for nucleotide data. Trees were visualized using the *ggtree* R package [43].

#### 2.3.5. Bayesian molecular clock analysis

Molecular clock analysis was performed using BEASTv2.6 [44]. We used a two steps clock strategy as in [45]: we first used fossil calibrations and a large dataset of outgroup sequences to estimate *F*. *sylvatica* origin, and then used these estimates to calibrate a second dataset centered on *F*. *sylvatica*.

We calibrated the molecular clock of the amino acid chloroplast dataset similarly to [46], using fossil evidence on the split between *Fagus* and all other genera (Maximum age: 82 Mya; Minimum age: 81 Mya; [47]) and that between the genera *Castanea* and *Castanopsis* (52 Mya; [48, 49]). Therefore, we set a root calibration at the *Fagus* node using a log-normal distribution (mean = 0.5; SD = 1.0; offset = 81.0; mean in real space = 81.5) and a monophyletic constraint at the node *Castanea*-*Castanopsis*, with another log-normal distribution (mean = 1.0; SD = 1.10; offset = 52.2; mean in real space = 53.2). We set a cp REV site model with gamma = 4, a relaxed-clock with a log-normal distribution, and a birth-death prior. The results of this first analysis were used to calibrate a second molecular clock, with the aim of dating the split between the two populations of *Fagus sylvatica* included in our study (ALP and APE). In particular, the median age estimates of the split between *Fagus sylvatica* and *Fagus crenata* (11.02 Mya, see Results) were used as root calibration, setting a normal distribution with mean = 11.02; SD = 1.0, and posing a monophyletic constraint on the *Fagus sylvatica* clade. We employed nucleotides and used a GTR site model with gamma = 4 and a relaxed-clock with a log-normal distribution. For the population prior, we set a Coalescent Bayesian Skyline model, as this has been shown to be particularly suitable when dealing with a mixture of intra- and inter-population data [50]. In all cases, we ran the analysis for 500 million generations, sampling and storing trees every 50,000 generations and assessing the convergence of chains using Tracer [51], making sure that all parameters showed Effective Sample Size (ESS) > 200. Maximum clade credibility trees were generated using TreeAnnotator, discarding the first 10% of the analysis (1000 trees) as burn-in. We visualized all timetrees using the *MCMCtreeR* package [52] and refined our figures in InkScape [53].

### 2.4. Whole-genome sequencing (WGS) analysis

#### 2.4.1. Reads mapping and SNP calling

Quality control of the reads was assessed with FASTQC version 0.11.8 [54]. Reads were then processed with fastp version 0.20.0 [55] for trimming and adapters removal; read tails with a mean Phred-quality score < 15 over a 4-bp sliding window were trimmed. Subsequently, the trimmed reads were mapped to the chromosome level *Fagus sylvatica* genome [25] (available at http://www.beechgenome.net) using the Burrows-Wheeler Aligner (BWA-MEM, v0.7.17; [56]) with “-R” flag for RG tag implementation. Alignments in sam format were sorted, marked for duplicates, indexed and compressed in BAM format using SAMTOOLS version 1.9 [57]; the mapped reads were used for downstream analysis and BAM files were validated using the ValidateSamFile tool of PICARD v2.25.4 [58]. The observed average depth of coverage was computed for each sample using the depth command of SAMTOOLS version 1.9. The previously available German (BHAGA) and Polish (JAMY) individuals, sequenced at higher coverage, were downsampled to a depth of coverage of 15x using the function DownsampleSam in PICARD tools, in order to facilitate comparison with the Italian samples.

Variant calling was performed using the Haplotype Caller (in -ERC GVCF mode) and GenotypeGVCFs tools in GATK v4.2.3.0 [59, 60]. We then filtered SNPs using GATK VariantFiltration tool, by excluding variants matching at least one of the following criteria: not a SNP, a significant Fisher strand test (FS > 60), a Variant Confidence/Quality by Depth (QD) < 2, a root mean square of the Mapping Quality (MQ) < 40, an MQRankSum < −12.5 or a significant read position bias (ReadPosRankSum < −8.0), depth of coverage (DP) < 0.25x or > 4x the mean coverage across samples. Lastly, only biallelic SNPs were retained, using the “—min-alleles 2” and “—max-alleles 2” selection flags in VCFtools [61].

#### 2.4.2. Variant annotation

The SNPs annotation was performed using SnpEff software 5.1 (https://pcingola.github.io/SnpEff/; [62]). Due to the lack of database for European beech among the prebuilt databases, we built a database using the *F*. *sylvatica* reference genome and its annotation list (Bhaga_genes.gff3) (http://thines-lab.senckenberg.de/beechgenome/data.html). The VCF file produced as described in 2.4.1 was used as the input file, and SnpEff annotated each SNP according to its genomic position (e.g., exons or introns, etc.) and predicted effect (missense, synonymous, etc.), producing a list of annotated variants, in which each variant was noted for its impact. Based on the definition of the sequence ontology (SO) terms (http://www.sequenceontology.org/) that SnpEff assigns as variant effects, we divided the SNPs into functionals and neutrals, creating two VCF files. We considered functional variants all those SNPs that were assigned the following SO terms: ’missense variant’, ’initiator codon variant’, ’start lost’, ’stop gained’, ’stop retained variant’, ’3 prime UTR variant’, ’5 prime UTR premature start codon gain variant’, ’5 prime UTR variant’, ’splice acceptor variant’, ’splice donor variant’ and ’splice region variant’. The following terms were considered for neutral variants classification: ’downstream gene variant’, ’intergenic region’, ’intron variant’, ’upstream gene variant’ and ’synonymous variant’. The SnpSift ’filter’ command was used to filter the annotated VCF file. Subsequently, neutral variants were further filtered out of all terms considered for the functional SNPs categories because of multiple effects that can be assigned to individual SNPs. Overall genetic diversity levels were estimated separately for the functional and neutral SNPs subsets (see the following section).

#### 2.4.3. Whole-genome genetic diversity estimates

In order to make sure that the trees sampled within each forest stand were not closely related, we estimated genome-wide pairwise relatedness coefficients (r) using the—make-rel command in PLINK 1.9 [63, 64]. The inbreeding coefficient (F) for each sample was computed using the -het option in PLINK, based on the observed and expected homozygous genotype counts.

For the two Italian populations, we estimated standard measures of genetic diversity: genome-wide heterozygosity (GW-het), nucleotide diversity (π), and Tajima’s D. Genome-wide (global) heterozygosity was calculated per sample using ANGSD v0.934 [65]. This is considered a better estimate than SNP-based heterozygosity proportion because it covers the whole genome [66]: briefly, the proportion of heterozygous sites is obtained for each individual genome by dividing the number of heterozygous sites (derived by the site frequency spectrum, SFS), by the total number of sites (i.e. the adjusted genome size; [65]). Population-based estimates of genome-wide heterozygosity for ALP and APE were then obtained by averaging the individual values. We used PIXY v1.0.0 to calculate unbiased nucleotide diversity, based on a VCF file that included invariant sites [67] and using non-overlapping sliding windows of 10 kb. Per site nucleotide diversity values were then plotted for each chromosome, to investigate general patterns along the genome and potential differences between populations. Tajima’s D was calculated using VCFtools [61], with non-overlapping sliding windows of 10 kb. Lastly, we estimated the overall neutral and functional genetic diversity levels in the Italian samples by computing the nucleotide diversity (π) and the average values of observed heterozygous sites (O-Het) from the neutral and functional SNPs subsets generated as described in the previous section. For the functional SNPs subset, we also provided separate estimates for the ALP and APE populations, for comparison.

#### 2.4.4. Linkage disequilibrium (LD) decay and runs of homozygosity (ROHs)

For a more in-depth characterization of whole-genome patterns of variation, we assessed linkage disequilibrium (LD) decay and runs of homozygosity (ROH) in the two Italian populations.

LD decay may reveal important information about a species’ demographic history and mating system [68]; moreover, different LD decay patterns in forest trees may render certain species better suited for some specific genetic studies (e.g., association mapping) than others [69]. LD decay along the beech genomes was calculated using PopLDdecay [70]; the LD decay curve was fitted using Generalized Additive Models (GAMs) by the gam function in the *mgcv* R package [71]. Due to the low sample size, the LD analysis was performed on all the Italian samples considered as a whole.

Runs of homozygosity (ROH) are contiguous homozygous segments of the genome, which can arise from the mating of related individuals and the subsequent transmission of identical haplotypes to the offspring [72, 73]. The R package *detectRUNS* [74] was used to detect ROH in each sample with a sliding-window method (function “slidingRUNS.run”) analogous to that implemented in PLINK [58, 68]. Briefly, ROH detection was determined in sliding windows of 10 SNPs, based on the following criteria: (i) the minimum number of SNPs in a ROH was 10; (ii) the minimum length of a ROH was 100 kb; (iii) the minimum SNP density was set to one SNP every 200 kb; (iv) a maximum gap of 1 Mb allowed between two consecutive homozygous SNP in a run; (v) a maximum of two missing and one heterozygous SNP were allowed in a window; and (vi) the window threshold of 0.05 was kept as default. We chose 100 kb as the minimum ROH length after testing different threshold values; the effect of choosing lower and higher minimum SNP density to call a ROH was also evaluated in a preliminary analysis.

#### 2.4.5. Whole-genome genetic differentiation and population structure

To examine topological relationships among all the European beech genomes in the dataset, we constructed a phylogenetic neighbor-joining tree using the *aboot* function from the R-package *poppr* [75], based on a pairwise identity-by-state (IBS) distance (i.e. Hamming distance; [76]) matrix calculated from the genome-wide SNPs using PLINK v1.9 [63, 64]. The distinctiveness of the two Italian populations, i.e. ALP and APE, was then evaluated using principal components analysis (PCA), implemented in PLINK v1.9 (—pca option) and plotted using the *tidyverse* collection of R packages [77]. An additional PCA analysis was also run including the German (BHAGA) and Polish (JAMY) individuals.

The level of genetic divergence between the two Italian populations was then assessed by estimating pairwise Nei’s GST [78] and Jost’s D [79], using the R package *vcfR* [80]. All the above-described analyses were restricted to a SNPs set having a minor allele frequency (MAF) ≥ 5% and linkage disequilibrium between variants of r^2^ < 0.50, since strongly linked markers and rare variants may introduce bias in population structure analysis from WGS data [81, 82]. We used PLINK v1.9 (—indep-pairwise function) to prune out loci in linkage disequilibrium (LD) using a sliding window size of 50 kb, step size of five loci, and r^2^ threshold of 0.5.

## 3. Results

### 3.1. Genomic diversity and differentiation of two European beech stands from the Italian peninsula (Alps and Apennines)

#### 3.1.1. Chloroplast and mitochondrial genomes diversity and differentiation

An average of 7,302,798 and 27,519,618 reads per individual were mapped to the chloroplast (cp) and mitochondrial (mt) reference genomes, with 915x and 237x average coverage, respectively. The proportion of mapped reads was higher for mt genomes (average 4.8%) than cp genomes (average 1.2%).

We identified a total of 20 SNPs and 54 InDels in the cp genomes of the 13 included individuals and 30 SNPs and 33 InDels in their mt genome. Overall nucleotide diversity was on average higher for cp genomes (NucDiv = 6.3 × 10^−5^) than for mt ones (NucDiv = 1.9 × 10^−5^). A total of 12 and 8 haplotypes were inferred for the cp and mt genomes, respectively, leading to a haplotype diversity (HapDiv) of 0.98 and 0.93, respectively. Almost all haplotypes related to the cp genomes were unique except for haplotype X, which was shared by two Apennine individuals, while one mt haplotype (II) was shared by three Apennine individuals and another haplotype (V) by two Alpine samples and FASYL29 (Germany). The lists of haplotypes detected in each sample at cpDNA and mtDNA are reported in Table 1; values for nucleotide and haplotype diversity for the two Italian populations (ALP and APE) are reported in Table 2.

**Table 1:** List of (a) cpDNA and (b) mtDNA haplotypes detected in the samples. Samples are ordered based on geographical provenance: APE, ALP (samples sequenced in the present study, from the Apennines and Alps, respectively) and the publicly available reference samples (see section 2.2): FSYL11 (Alps), FASYL29 (Northern Germany), BHAGA (Central Germany) and JAMY (Poland); haplotypes (hap) are grouped by clade (cl); only cpDNA genome was available for FSYL11.

**(a) cpDNA genome**														
**cl**	**hap**	**APE1**	**APE2**	**APE3**	**APE4**	**APE5**	**ALP1**	**ALP2**	**ALP3**	**ALP4**	**FSYL 11**	**FASYL 29**	**BHAGA**	**JAMY**
2	I										x			
2	II											x		
2	III						x							
2	IV							x						
2	V								x					
2	VI									x				
2	VII													x
2	VIII												x	
1	IX	x												
1	X		x	x										
1	XI				x									
1	XII					x								
**(b) mtDNA genome**														
**cl**	**hap**	**APE1**	**APE2**	**APE3**	**APE4**	**APE5**	**ALP1**	**ALP2**	**ALP3**	**ALP4**		**FASYL 29**	**BHAGA**	**JAMY**
1	I	x												
1	II		x	x		x								
1	III				x									
2	IV						x							
2	V							x	x			x		
2	VI									x				
2	VII													x
3	VIII												x	

**Table 2:** Levels of genetic diversity for the two sequenced Italian populations. For samples from ALP (Val di Cembra, Eastern Italian Alps) and APE (Maresca, Northern Apennines), population-based genetic diversity estimates were computed for organellar and nuclear genomes (no. hap = Number of haplotypes; hapDiv = haplotype diversity; nucDiv = nucleotide diversity; D = Tajima’s D; GW-het = genome-wide heterozygosity; π = genome-wide nucleotide diversity).

Pop	mtDNA genome				cpDNA genome				Nuclear genome		
	No. hap	hapDiv	nucDiv	Tajima’s D	No. hap	hapDiv	nucDiv	Tajima’s D	GW-Het	π	Tajima’s D
ALP	3	0.83	0	-8.52	4	1	7.36 × 10^−6^	-5.47	0.0167	0.0034	0.5096
APE	3	0.7	1.58 × 10^−6^	-4.96	4	0.9	2.52 × 10^−6^	-6.44	0.0179	0.0037	0.4975

**Fig 2:**
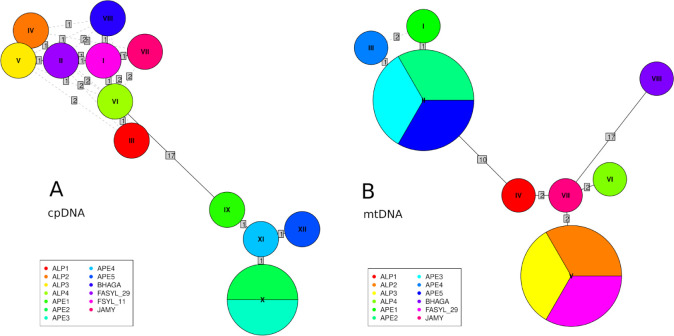
Minimum spanning networks of haplotypes based on the whole (A) cpDNA and (B) mtDNA genomes. Each haplotype is represented by a circle with size proportional to the number of individuals and is colored according to the individuals harboring it (see legend); numbers above branches indicate the number of mutation events between two haplotypes; dashed segments represent alternative links between haplotypes. The networks were generated using the R package *pegas*.

In the inferred cpDNA network (Fig 2A), two groups are recognizable: the samples from Apennines (APE) on one side (clade 1) and all the other samples on the other (clade 2), including the Alpine samples sequenced in the present study (ALP) and the publicly available chloroplast genomes retrieved from NCBI (see section 2.2): FSYL11 (Alps), FASYL29 (Germany), BHAGA (Germany), JAMY (Poland). The inferred mtDNA haplotype network (Fig 2B) separates three main groups: clade 1, including the samples from the Apennines (APE); clade 2, including the Alpine samples sequenced in the present study (ALP), the German sample FASYL29 and the Polish sample JAMY; and clade 3, including only the German sample BHAGA.

#### 3.1.2. Diversity and differentiation based on the nuclear genome

We produced a total of ~ 617.15 million reads which uniquely mapped to the European beech nuclear reference genome. The mean depth of coverage for the four samples from Alps (ALP) was 15.9x while the mean depth of coverage for the five samples from the Apennines (APE) was 16.6x. After VCF filtering, a total of 1,261,484 biallelic SNPs were retained across 11 individuals (also including the two Central European reference samples, BHAGA and JAMY, downsampled at 16x; see section 2.1). The final mean depth of coverage, No. of biallelic SNPs and mean SNP density are reported for each sample in S1 Table.

Relatedness coefficients (r) between the individuals sampled within each forest stand resulted to be negative in all pairwise comparisons, indicating unrelated samples [83] (ALP: average -0.37 ± 0.02 SD; APE: average -0.30 ± 0.02 SD; see S4 Table). Individual inbreeding coefficient (F) ranged from 0.54 to 0.71, with comparable average values in the two Italian populations (mean F = 0.59 for ALP; mean F = 0.60 for APE). The two Italian populations showed similar levels of genome-wide genetic diversity (Table 2), with slightly higher values of average genome-wide heterozygosity (GW-het; individual values reported in S1 Table) and nucleotide diversity (π) in the APE (GW-het = 0.0179; π = 0.0037) compared to ALP population (GW-het = 0.0167; π = 0.0034). The sliding window plot of the nucleotide diversity per site (π) highlighted a major anomaly on chromosome 11 (S1 Fig), where in a stretch of about 2 Mb (from about Mb 16 to Mb 18), repeated consecutive π values near to 0 were observed, around a peak of about 0.002. This region perfectly matches the one described in [25], where multiple and very long insertions of both chloroplast and mitochondrial DNA were observed.

**Fig 3:**
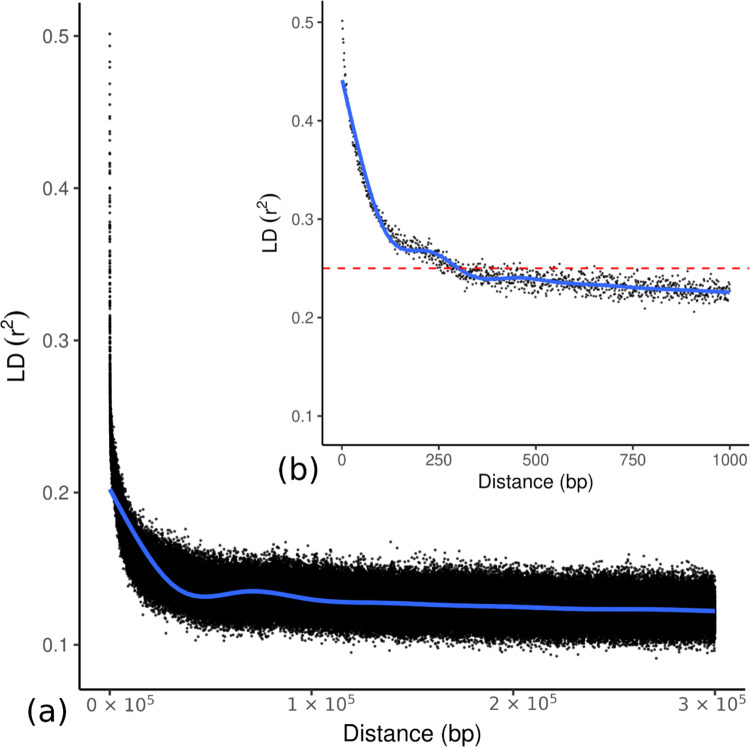
Linkage disequilibrium (LD) decay. (a) across the whole genome; (b) zoom for distances ≤ 1000 bp. The plot represents the average genome-wide LD decay in the samples from the two Italian stands (ALP and APE). The values on the X-axis represents the physical distance in (bp) and the Y-axis represents the average squared correlation, r^2^, between SNP alleles. The solid blue line depicts the LD decay curve fitted using Generalized Additive Models (GAMs); the dashed red line indicates the half-decay distance (r^2^ = 0.25).

Results of the LD analysis on all the nine Italian samples showed a rapid decay of r^2^ values: the half-decay distance, i.e. the distance at which LD is half of its maximum value was found to be of about 250 bp on average (corresponding to r^2^ = 0.25); however, r^2^ values remain > 0.2 at 1000 bp and the fitted LD decay curve approaches 0.1 slowly (Fig 3).

No significant differences between the two Italian populations emerged from the runs of homozygosity (ROH) analysis. The average number of detected ROHs was 355 ± 22 in the APE population, 354 ± 81 in the ALP populations; these values were not so different from those detected in the two Central European samples (BHAGA: 400; JAMY: 452). In general, the length of the detected ROHs was very low, with only five ROHs > 0.5 Mb (one detected in a sample from ALP, three in samples from APE and one in BHAGA) and no ROHs > 1 Mb (max ROH length: 0.59 Mb). Results remained consistent even using different values of SNP density to call a ROH.

**Fig 4:**
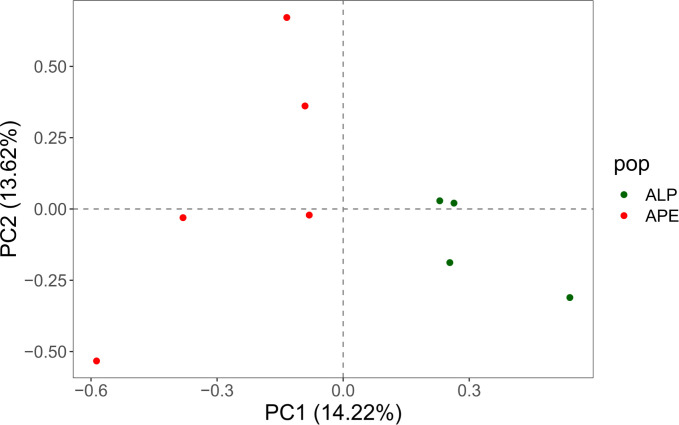
Principal component analysis (PCA) plot of the samples from the two Italian stands. The analysis is based on the 38,271 genome-wide SNPs dataset; individuals are plotted on the biplot of the first two principal components.

After filtering for MAF ≥ 5% and LD pruning (r2 < 0.50; see section 2.4.5), 38,271 SNPs were retained for genetic differentiation and population structure analyses. In the whole genome-based NJ tree (S2 Fig), the four samples from the Italian Alps (ALP) were grouped in the same cluster, together with the German sample (BHAGA), while a less homogeneous structure emerged for the five samples from the Apennines (APE), with the Polish sample (JAMY) clustering closer to them. A principal component analysis (PCA) performed on the filtered SNPs highlighted a similar situation: the PCA plot (S3 Fig) showed an apparent, although not complete, separation between ALP and APE samples along the first two principal components (PC1 and PC2), with BHAGA closer to the ALP samples along PC1, while JAMY falling in an intermediate position. Points corresponding to APE samples are more spread out in the plot compared to ALP, exhibiting a larger variance, consistently with the result of the tree. A PCA performed with only the Italian samples (Fig 4) highlighted a more clear separation between ALP and APE, confirming more variability in the APE samples. Pairwise GST (0.157) and Jost’s D (0.089) values indicated a clear genetic differentiation between the two Italian populations, reflecting the above described patterns.

### 3.2. Phylogenetic analysis: Ancient split between the Alpine and Apennine populations

We employed phylogenetic methods to understand how and when the two populations differentiated. For the molecular clock analysis, we were constrained by the absence of reliable substitution rates for both mitochondria and chloroplasts in this taxon. To overcome this limitation, we followed a strategy based on a 2-steps molecular clock analysis, using the estimated divergence times between the *Fagus* genus and the other groups of Fagaceae of a first long-scale reconstruction to calibrate a second analysis on a shorter timescale.

**Fig 5:**
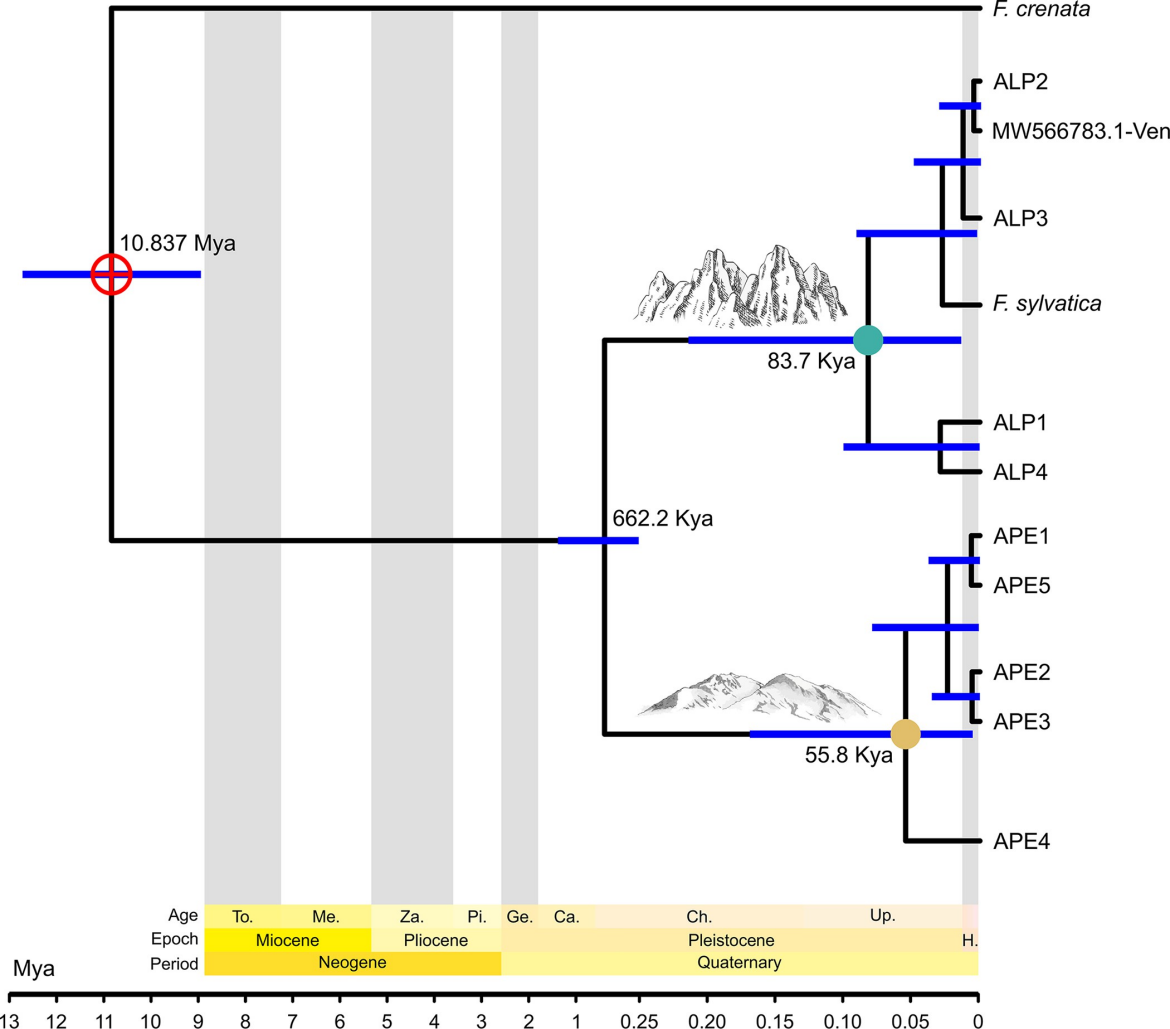
Bayesian molecular clock analysis of APE (light brown dot) and ALP (light blue dot) populations using cpDNA. The references of *F*. *crenata* (NC_041252.1) and two individuals of *F*. *sylvatica* (NC_041437.1 and MW566783.1) were included in the analysis. Median height values of the deepest nodes in the tree are shown close to each node; the scale of the tree in the figure changes at 1 Mya. The split between *F*. *crenata* and *F*. *sylvatica* (the node highlighted with a crossed circle) was calibrated using results of the clock shown in S5 Fig.

For the mitochondrial dataset, we were unable to perform a meaningful phylogenetic and clock analysis, as only five Fagaceae mitochondrial genomes were available in public databases at the time of the analysis (three of which belonging to *F*. *sylvatica*). This shows the necessity of improving sequencing effort of mitochondrial genomes of this taxon. Our newly sequenced mitochondrial genomes were almost identical in their coding regions, with polymorphisms concentrated in the non-coding part of the genome, which were sufficient to discriminate between the Alpine and Apennine populations (S4 Fig) but prevented a robust clock strategy based on the analysis of coding sequences. Therefore, we used chloroplast data to reconstruct a maximum-likelihood phylogeny and a calibrated Bayesian phylogeny (S5 Fig) of a dataset including different species of Fagaceae. To minimize compositional biases, we employed a set of 57 single copy orthologous genes (see section 2.3.2). Trees obtained with these two different approaches yielded the same topological structure. In both cases, *Fagus crenata* was the sister species of *Fagus sylvatica* (bootstrap supports in the maximum-likelihood tree for this node were low, but very high for all the other groups). We dated the split between these two taxa at a median of 11.02 Mya (Mean: 11.81; 22.4–2.4 Mya 95% HPD; S5 Fig). We used the topology and the date estimates of this clock analysis to calibrate a second molecular clock (Fig 5), with the aim of dating the splits between our samples.

Bayesian molecular clock analysis suggests that the split between the Alpine and Apennine populations occurred at a median of 662.2 Kya, between the Günz and the Riss glaciations (Mean: 757.6 Kya; 95% HPD: 1.38 Mya– 258.1 Kya). The last common ancestors of the individuals belonging to the Alpine (Median: 83.7 Kya, Mean: 103.3 Kya; 95% HPD: 218.6 Kya– 13.9 Kya) and Apennine population (Median: 55.8 Kya, Mean: 72.3 Kya; 95% HPD: 172.7 Kya– 0.54 Kya), respectively, are both dated in the Würm glaciation. The same topology, with a sharp separation between ALP and APE populations, was retrieved across all datasets and methods. The estimate for the split between *F*. *crenata* and *F*. *sylvatica* in the second calibration analysis (Median: 10.837 Mya; Mean: 10.838; 95% HPD: 12.84–8.83 Mya) resulted to be highly consistent with the one calculated in the first analysis (Median: 11.02; Mean: 11.81; 22.4–2.4 Mya 95% HPD), with only a slight difference which could be expected due to the inherent nature of the Bayesian analysis and the different priors used to calibrate the clock.

### 3.3. Variant annotation and functional characterization

The average SNP rate over all chromosomes was 1 every 424 bases. SNPs distribution on each chromosome was analyzed to reveal the most polymorphic regions. The highest number of variants (355,656) was found on chromosome 1, as expected based on chromosome length. The highest variant rate (region length bp / number of variants) was found on chromosome 4 (851), and the lowest (328) on chromosome 5.

By analyzing the annotations of all the SNPs (1,261,484) deriving from the whole-genome sequencing of the Italian samples, we found a total of 2,783,020 predicted effects. According to the type of change and its effect, only 9135 (0.3%) SNPs were predicted to have a high impact (e.g., stop codon gaining, frameshift), whereas 222,199 (8%) represent SNPs with moderate to low impact effects (e.g., non-synonymous change, synonymous coding/start/stop). In addition, SnpEff also classified as missense variant 132,606 SNPs, as stop gained 6611 SNPs, and 79,861 SNPs as silent functional classes (S3 Table).

The highest number of SNPs identified were located in the upstream (27.65%) and downstream (26.26%) gene regions. We found 778,498 (27.9%) SNPs in intergenic regions (S6 Fig) and 486,933 (18.2%) SNPs within genes, including intronic regions (8.2%) and 3’ and 5’ prime variants (1.7%). Most of these nucleotide changes can be classified as transitions, with a transition/transversion ratio (Ts/Tv ratio) of 2.1. Based on the effects type (see section 2.4.2), 188,600 variants could be considered functional, while 1,072,884 were classified as neutral.

Results of the genetic diversity analysis performed separately on the neutral and functional SNPs subsets for the overall Italian samples highlighted a prevalence of neutral variation, as expected; however, functional variation represented a numerically important component, with an average value of observed heterozygous sites of 11,902, only about six times lower than the average values of observed heterozygous site observed in the neutral SNPs subset (68,416) (average nucleotide diversity: 1.1 × 10^−4^ in the functional SNPs subset and 2.8 × 10^−4^ in the neutral SNPs subset, respectively). No differences were observed in the functional diversity levels of the two Italian populations, ALP and APE, both in terms of observed heterozygous sites and nucleotide diversity (data not shown).

## 4. Discussion

### 4.1. Genome-wide diversity of European beech in the Italian peninsula

Identifying, quantifying, and understanding genome-wide patterns of genetic diversity is of crucial importance for forest trees under climate change [84], and particularly for foundation species occupying wide and climatically variable geographic areas, such as *Fagus sylvatica*. Our nuclear, chloroplast and mitochondrial genomes analysis of nine European beech trees, four of them sampled in a forest stand located in the Italian Alps (Trentino region; ALP) and five from the central Apennines (Tuscany region; APE), provided a first genome-wide overview of genetic diversity for this important tree species in the Italian peninsula, close to the southern margin of its distribution.

Genetic diversity levels were comparable in the two sampled Italian forest stands, ALP and APE, at both organellar and nuclear genomes. At organellar genomes, the same number of haplotypes was detected in ALP and APE: four at cpDNA and three at mtDNA, reflecting a relatively high diversity despite the low sample size (four and five individuals, respectively). At nuclear genomes, no significant differences were detected in terms of mean heterozygosity, nucleotide diversity and Tajima’s D (Table 2). Our genome-wide nucleotide diversity estimates (ALP: 3.4 × 10^−3^; APE: 3.7 × 10^−3^) were higher, albeit of the same magnitude, than those observed in previous studies in the European beech in other European areas [85, 86].

A rapid genome-wide LD decay was highlighted, as expected for an outcrossing forest tree species, with the correlation coefficient decaying to half of its maximum value (r^2^ = 0.25) at about 250 bp; however, after this major drop LD was found to decrease relatively slowly. Both findings are in line with the results reported by [85] for a French population of the species based on a set of 58 genes, and highlight a global LD decay slower than expected, compared with other broad-leaved tree species, e.g., *Populus tremula* [87] or *Eucalyptus globulus* [88]. According to LD estimates, the extent of ROHs was rather small (with no significant differences among the two populations), with the exception of a region of about 2 Mb on chromosome 11, where nucleotide diversity was close to zero (S1 Fig). The location of this region perfectly corresponds to an anomaly in the European beech genome, previously identified by [25] and characterized by long and multiple insertions of both chloroplast and mitochondrial DNA: we believe that this is the most likely explanation for the recorded local pattern of diversity.

Functional annotation of SNPs identified at the genome-wide level was performed, based on their genomic locations. As there are not many published studies on SNPs effects in *Fagus sylvatica*, we attempted to compare our variant annotation data obtained by a WGS approach with a previous study [89] that used three different genotyping methods (GBS, RADSeq and ddRAD) for SNPs marker discovery, although there is a marked difference in the number of samples examined. Our SNPs distribution (S3 Table) is more comparable to GBS results, where the majority of SNPs (> 75%) are associated with non-genic sites, than those of ddRAD (66.4% of variants in non-genic regions). In particular, our data are more divergent with respect to the RADSeq results (more than 50% of SNPs in genetic regions), especially when comparing the number of variants annotated for each effect type. Moreover, WGS approach allows us to provide a more exhaustive variants description, which also includes a large portion of SNPs located in the intergenic regions (about 28%) compared to RAD methods (only from 7 to 10%). This result could not only be due to a reduced genome representation, but the identification of SNPs in different regions often depends on the use and choice of restriction enzymes [90].

The low number of individuals sampled, limited us from performing further analysis at the population level by exploiting variants with different coding effects. However, we believe that our list of annotated SNPs may represent a resource for the community, in particular for future studies on European beech that combine variant annotation and gene expression analysis. These data can be used in much broader studies and, focusing mainly on high-impact variations, may provide important insights into the function of genes involved into the adaptation mechanism of this species [91–93].

### 4.2. Strong and ancient split between Alps and Apennines

The cpDNA and mtDNA haplotype networks (Fig 2) clearly showed that samples from the Alps (ALP) and Apennines (APE) form two distinct, well differentiated clades. The Italian sample FSYL11, for which a publicly available chloroplast genome was available (see section 2.2) and included in the cpDNA network, grouped in the same clade with our Alpine samples: this is not surprising, since FSYL11 is an individual from the Veneto region, in the eastern Alps, not far from the Trentino region where our Alpine individuals were sampled. As largely expected, the differentiation of mt genome was lower than that of cp, in spite of its larger genome size (ca 500 kb vs 167 kb).

The separation between Alps and Apennines was confirmed by the analysis of the nuclear genomes: the PCA with the Italian samples (Fig 4) and the NJ tree (S2 Fig) showed that all individuals from the ALP population grouped together in a relatively homogeneous group, which appears separated from the cluster formed by the APE samples, although the latter were more scattered in the PCA space as well as in the tree. The separation between ALP and APE was also quantitatively confirmed by the two widely used indices GST and Jost’s D, with values of 0.16 and 0.09, respectively, pointing to a clear genomic differentiation between the two Italian populations, reflecting the patterns described by the NJ tree and the PCA plot.

We deliberately refrain from discussing the differentiation between the German (BHAGA and FASYL29) and Polish (JAMY) samples, their relationships with the Italian populations and the apparent incongruence between nuclear and organellar trees for BHAGA (see Results). These European areas were not covered here, being out of the scope of the present study, and only a few genomes were publicly available (considering the nuclear genome, only for the BHAGA and JAMY individuals); these regions are, moreover, characterized by supposedly complex evolutionary history, with a potential distinct glacial refugium hypothesized for Central-Eastern Europe and evidence for admixture among different lineages north of the Alps [7–10].

Although we are aware that the reduced sample size of our survey prevents us from reconstructing a comprehensive evolutionary scenario for the species in the Italian peninsula, our analysis based on complete chloroplast, mitochondrial and nuclear genomes not only confirmed the genetic separation between the Alpine and Apennine populations, already identified by previous studies and congruent with a scenario of different glacial microrefugia in the Italian peninsula [7, 11–13], but also allowed us, for the first time, to derive a reliable temporal estimate for the split. By adopting a 2-steps molecular clock analysis, we first inferred the divergence time between *F*. *sylvatica* and *F*. *crenata* cpDNA genomes (Fig 5); then this estimate was used to calibrate a second analysis centered on *F*. *sylvatica*, which ultimately allowed us to date the split among the two Italian populations (Fig 5). The adoption of this strategy, which has been previously used for example to estimate the divergence time of polar bear [45], allowed us to overcome one of the major obstacles of inferring divergence estimates: the lack of reliable rates of substitution and/or of fossil calibrations. We could not replicate this strategy with mitochondrial data, as entire mt genomes within the Fagaceae are available just for three species. Moreover, our newly sequenced mitochondrial genomes were almost all identical in their coding regions: this is coherent with the biology of plant mitochondrial genomes that evolve at a slower pace than the chloroplast and nuclear ones, although showing large genome rearrangements (e.g., inversions, translocations, duplications, losses; [94]. In our chloroplast phylogeny we used a set of 57 single copy ortholog genes: we obtained a robust tree showing that *F*. *crenata* is the sister species of *F*. *sylvatica*. These results are in line with [95], but different than those of a recent analysis using full chloroplast genomes [96, 97]. We suggest that some previous results may have been misled by the effect of high saturation, which diminishes excluding fast evolving regions of the genome and working with well conserved genes, as we did. Our molecular clock estimate for the split between ALP and APE cp genome was at a median of 662.2 Kya (Mean: 757.6 Kya; 95% HPD: 1.38 Mya– 258.1 Kya; Fig 5), approximately between the Calabrian and the Chibanian Stages, between the Günz and the Riss glaciations: therefore, well before the last glacial cycle (115 to 11 kya; [98]. This finding is in line with the conclusion of [7] who, according to palynological and genetic (not genomic) results, hypothesized that European beech might have persisted in the same refugial areas for at least two interglacial–glacial cycles. According to these authors, one of these refugia was located in central-southern Italy, thus corresponding to the central and southern ridges of the Apennines. Finally, they claimed that the observed genetic divergence was probably generated in a time span of hundreds of thousands of years, and our results seem to confirm this hypothesis. Evolutionary estimates of divergence time between populations on the Balkan and Italian peninsulas have led to backdating to before the last glacial period for other forest species such as silver fir [99, 100] and *Ulmus laevis* [101]. Here it is worth noting that a similar old divergence among main genetic lineages, predating the Last Glacial Maximum, has been proposed for many other plant and animal taxa in the Italian peninsula (for a meta-analysis, see [102]).

### 4.3. Conclusions and conservation implications

Our results mark a significant improvement of the genomic information of *Fagus sylvatica*, a keystone and economically important tree species, in one of the most southern areas of its distribution: the Italian peninsula.

Although being a widely distributed deciduous broadleaf temperate tree, *Fagus sylvatica* is currently experiencing drought-induced growth decline and increasing mortality due to climate change, particularly at its southern edge, with predicted serious ecological and economic consequences [5, 6, 103]. On the other hand, recent studies are also showing that marginal populations of the species may exhibit different responses to drought and other climatic stress [22, 104, 105]. Therefore, deciphering the response of marginal beech populations to climate change is essential for adapting the current forest management strategies, and a proper and deep knowledge of gene pools in these areas may foster more rigorous and well-grounded adaptation studies.

Here, we demonstrated that the Alpine and Apennine populations of *Fagus sylvatica* experienced a long history of separation, predating the last glaciation. As a consequence, the genomic diversity of the species in the Italian peninsula has been shaped by repeated interglacial–glacial cycles, possibly leading to distinct local adaptation trajectories: this scenario seems not to be unlikely, also considering the overall high levels of functional variation detected in the Italian samples; however more specific studies are needed to confirm this hypothesis.

To conclude, our findings clearly showed the informative power of whole-genome data for the identification and management of in situ genetic conservation units for forest trees.

## Supporting information

S1 Table List of the analyzed European beech nuclear genomes. For each sample, the average depth of coverage, No. of SNPs and SNP density are reported (inferred from the filtered VCF files), together with individual-based estimates of genome-wide diversity (F = inbreeding coefficient computed using PLINK; O(Hom) = Observed number of homozygous genotypes; E(Hom) = Expected number of homozygous genotypes).
(DOCX)

S2 Table List of all Fagaceae chloroplast and mitochondrial genomes used for phylogenetic inference. The annotated proteomes correspondent to each accession were downloaded from the NCBI website.
(DOCX)

S3 Table Functional annotations of the potential SNP effects. Summary of SnpEff annotation with the number of SNPs found in each effect type category.
(DOCX)

S4 Table Pairwise relatedness coefficients (r) between the individuals sampled within each forest stand (ALP and APE). (DOCX)

S5 Table GenBank accession numbers GenBank of the sequences of the chloroplast and mitochondrial genomes produced in the present study. (DOCX)

S1 Fig Per-site genome-wide nucleotide diversity. The plot shows the results of a sliding window analysis (window length = 10 kb) of nucleotide diversity per site (π) on chromosome 11, where large organellar genome insertions (from about Mb 16 to Mb 18) had been identified in a previous study [25].
(PDF)

S2 Fig Neighbor-joining tree of all the European beech samples included in the study. The analysis is based on pairwise identity-by-state (IBS) distance calculated from the 38,271 genome-wide SNPs dataset (after filtering for MAF ≥ 5% and LD pruning at r^2^ < 0.50).
(PDF)

S3 Fig Principal component analysis (PCA) plot of all the samples included in the analysis, based on the 38,271 genome-wide SNPs dataset. Individuals are plotted on the biplot of the first two principal components; the dataset includes nine Italian samples from the Alpine (ALP) and Apennine (APE) populations, one German (BHAGA) and one Polish sample (JAMY).
(PDF)

S4 Fig Maximum-likelihood phylogeny of the full mtDNA genomes of ALP and APE populations. Mitochondrial data show a sharp separation between the Alpine and the Apennine populations (these two clades are indicated by dots of different colors at the node), with very high bootstrap values. The two clades cannot be further divided into subclades reliably, as shown by the very low bootstrap supports inside these two groups. The tree was midpoint rooted. A scale bar indicating the substitutions per site is indicated below the tree.
(PDF)

S5 Fig Bayesian molecular clock analysis of Fagaceae cpDNA. Big and crossed red circles indicate fossil calibrated nodes. Full red circles indicate nodes for which posterior probabilities were below 60. Blue bars show the 95% highest posterior densities (HPDs) of the date estimates, representing the shortest interval on the posterior density of age estimates for a 95% confidence level.
(PDF)

S6 Fig Number of large-effect SNPs in the genome of *F*. *sylvatica* Italian populations (ALP and APE). SNPs classification according to their effect and distribution across genomic regions. The X-axis displays the variants number, and the Y-axis the different regions.
(PDF)

## References

[1] Packham JR, Thomas PA, Atkinson MD, Degen T. Biological Flora of the British Isles: *Fagus sylvatica*. J Ecol. 2012;100: 1557–1608. doi: 10.1111/J.1365-2745.2012.02017.X

[2] Pluess AR, Frank A, Heiri C, Lalagüe H, Vendramin GG, Oddou-Muratorio S. Genome–environment association study suggests local adaptation to climate at the regional scale in *Fagus sylvatica*. New Phytol. 2016;210: 589–601. doi: 10.1111/NPH.13809 26777878

[3] Csilléry K, Lalagüe H, Vendramin GG, González-Martínez SC, Fady B, Oddou-Muratorio S. Detecting short spatial scale local adaptation and epistatic selection in climate-related candidate genes in European beech (*Fagus sylvatica*) populations. Mol Ecol. 2014;23: 4696–4708. doi: 10.1111/MEC.12902 25156570

[4] Geßler A, Keitel C, Kreuzwieser J, Matyssek R, Seiler W, Rennenberg H. Potential risks for European beech (*Fagus sylvatica* L.) in a changing climate. Trees—Struct Funct. 2007;21: 1–11. doi: 10.1007/S00468-006-0107-X

[5] Gárate-Escamilla H, Hampe A, Vizcaíno-Palomar N, Robson TM, Benito Garzón M. Range-wide variation in local adaptation and phenotypic plasticity of fitness-related traits in *Fagus sylvatica* and their implications under climate change. Glob Ecol Biogeogr. 2019;28: 1336–1350. doi: 10.1111/GEB.12936

[6] Martinez del Castillo E, Zang CS, Buras A, Hacket-Pain A, Esper J, Serrano-Notivoli R, et al. Climate-change-driven growth decline of European beech forests. Commun Biol. 2022;5: 163. doi: 10.1038/s42003-022-03107-3 35273334 PMC8913685

[7] Magri D, Vendramin GG, Comps B, Dupanloup I, Geburek T, An Gömöry DS, et al. A new scenario for the Quaternary history of European beech populations: palaeobotanical evidence and genetic consequences. New Phytol. 2006;171: 199–221. doi: 10.1111/j.1469-8137.2006.01740.x 16771995

[8] Postolache D, Oddou-Muratorio S, Vajana E, Bagnoli F, Guichoux E, Hampe A, et al. Genetic signatures of divergent selection in European beech (*Fagus sylvatica* L.) are associated with the variation in temperature and precipitation across its distribution range. Mol Ecol. 2021;30: 5029–5047. doi: 10.1111/MEC.16115 34383353

[9] Meger J, Ulaszewski B, Vendramin GG, Burczyk J. Using reduced representation libraries sequencing methods to identify cpDNA polymorphisms in European beech (*Fagus sylvatica* L). Tree Genet Genomes. 2019;15: 1–9. doi: 10.1007/S11295-018-1313-630546292

[10] Stefanini C, Csilléry K, Ulaszewski B, Burczyk J, Schaepman ME, Schuman MC. A novel synthesis of two decades of microsatellite studies on European beech reveals decreasing genetic diversity from glacial refugia. Tree Genet Genomes. 2023;19. doi: 10.1007/s11295-022-01577-4 36532711 PMC9744708

[11] Magri D. Patterns of post-glacial spread and the extent of glacial refugia of European beech (*Fagus sylvatica*). J Biogeogr. 2008;35: 450–463. doi: 10.1111/j.1365-2699.2007.01803.x

[12] Vettori C, Vendramin GG, Anzidei M, Pastorelli R, Paffetti D, Giannini R. Geographic distribution of chloroplast variation in Italian populations of beech (*Fagus sylvatica* L.). Theor Appl Genet. 2004;109: 1–9. doi: 10.1007/S00122-004-1609-9 15014873

[13] Demesure B, Comps B, Petit RJ. Chloroplast DNA Phylogeography of the Common Beech (*Fagus sylvatica* L.) in Europe. Evolution (N Y). 1996;50: 2515. doi: 10.2307/2410719 28565658

[14] Leonardi S, Menozzi P. Genetic variability of *Fagus sylvatica* L. in Italy: the role of postglacial recolonization. Heredity. 1995;75:35–44. doi: 10.1038/hdy.1995.101

[15] De Lafontaine G, Ducousso A, Lefèvre S, Magnanou E, Petit RJ. Stronger spatial genetic structure in recolonized areas than in refugia in the European beech. Mol Ecol. 2013;22: 4397–4412. doi: 10.1111/mec.12403 23980761

[16] Meger J, Ulaszewski B, Burczyk J. Genomic signatures of natural selection at phenology-related genes in a widely distributed tree species *Fagus sylvatica* L. BMC Genomics. 2021;22: 1–20. doi: 10.1186/s12864-021-07907-5 34332553 PMC8325806

[17] Cuervo-Alarcon L, Arend M, Müller M, Sperisen C, Finkeldey R, Krutovsky K V. A candidate gene association analysis identifies SNPs potentially involved in drought tolerance in European beech (*Fagus sylvatica* L.). Sci Rep. 2021;11: 1–15. doi: 10.1038/s41598-021-81594-w 33504857 PMC7840767

[18] Pfenninger M, Reuss F, Kiebler A, Schönnenbeck P, Caliendo C, Gerber S, et al. Genomic basis for drought resistance in european beech forests threatened by climate change. Elife. 2021;10: 1–17. doi: 10.7554/eLife.65532 34132196 PMC8266386

[19] Capblancq T, Morin X, Gueguen M, Renaud J, Lobreaux S, Bazin E. Climate-associated genetic variation in *Fagus sylvatica* and potential responses to climate change in the French Alps. J Evol Biol. 2020;33: 783–796. doi: 10.1111/jeb.13610 32125745

[20] Rose L, Leuschner C, Köckemann B, Buschmann H. Are marginal beech (*Fagus sylvatica* L.) provenances a source for drought tolerant ecotypes? Eur J For Res. 2009;128: 335–343. doi: 10.1007/s10342-009-0268-4

[21] Kreyling J, Buhk C, Backhaus S, Hallinger M, Huber G, Huber L, et al. Local adaptations to frost in marginal and central populations of the dominant forest tree *Fagus sylvatica* L. as affected by temperature and extreme drought in common garden experiments. Ecol Evol. 2014;4: 594–605. doi: 10.1002/ece3.971 25035801 PMC4098140

[22] Wang F, Israel D, Ramírez-Valiente JA, Sánchez-Gómez D, Aranda I, Aphalo PJ, et al. Seedlings from marginal and core populations of European beech (*Fagus sylvatica* L.) respond differently to imposed drought and shade. Trees—Struct Funct. 2021;35: 53–67. doi: 10.1007/s00468-020-02011-9

[23] Koboldt DC, Steinberg KM, Larson DE, Wilson RK, Mardis ER. The next-generation sequencing revolution and its impact on genomics. Cell. 2013;155: 27–38. doi: 10.1016/j.cell.2013.09.006 24074859 PMC3969849

[24] Mishra B, Gupta DK, Pfenninger M, Hickler T, Langer E, Nam B, et al. A reference genome of the European beech (*Fagus sylvatica* L.). Gigascience. 2018;7: 1–8. doi: 10.1093/GIGASCIENCE/GIY063 29893845 PMC6014182

[25] Mishra B, Ulaszewski B, Meger J, Aury J-M, Bodénès C, Lesur-Kupin I, et al. A chromosome-level genome assembly of the European beech (*Fagus sylvatica*) reveals anomalies for organelle DNA integration, repeat content and distribution of SNPs. Front Genet. 2022;12: 2748. doi: 10.3389/FGENE.2021.691058/BIBTEX PMC8862710 35211148

[26] Radwan J, Babik W. The genomics of adaptation. Proc R Soc B Biol Sci. 2012;279: 5024–5028. doi: 10.1098/rspb.2012.2322 23097510 PMC3497254

[27] Allendorf FW, Hohenlohe PA, Luikart G. Genomics and the future of conservation genetics. Nat Rev Genet 2010 1110. 2010;11: 697–709. doi: 10.1038/nrg2844 20847747

[28] Migliore J, Kaymak E, Mariac C, Couvreur TLP, Lissambou BJ, Piñeiro R, et al. Pre-Pleistocene origin of phylogeographical breaks in African rain forest trees: new insights from *Greenwayodendron* (Annonaceae) phylogenomics. J Biogeogr. 2019;46: 212–223. doi: 10.1111/JBI.13476

[29] Caudullo G, Welk E, San-Miguel-Ayanz J. Chorological maps for the main European woody species. Data Br. 2017;12: 662–666. doi: 10.1016/j.dib.2017.05.007 28560272 PMC5435575

[30] Mader M, Liesebach H, Liesebach M, Kersten B. The complete chloroplast genome sequence of *Fagus sylvatica* L. (Fagaceae). Mitochondrial DNA Part B Resour. 2019;4: 1818–1819. doi: 10.1080/23802359.2019.1612712

[31] Mader M, Schroeder H, Schott T, Schöning-Stierand K, Montalvão APL, Liesebach H, et al. Mitochondrial genome of *Fagus sylvatica* L. as a source for taxonomic marker development in the Fagales. Plants. 2020;9: 1–19. doi: 10.3390/PLANTS9101274 32992588 PMC7650814

[32] Dierckxsens N, Mardulyn P, Smits G. NOVOPlasty: De novo assembly of organelle genomes from whole genome data. Nucleic Acids Res. 2017;45. doi: 10.1093/nar/gkw955 28204566 PMC5389512

[33] Seemann T. Snippy: rapid haploid variant calling and core genome alignment. 2015. Available: https://github.com/tseemann/snippy

[34] Katoh K, Standley DM. MAFFT multiple sequence alignment software version 7: improvements in performance and usability. Mol Biol Evol. 2013;30: 772–780. doi: 10.1093/molbev/mst010 23329690 PMC3603318

[35] Mishra B, Ulaszewski B, Ploch S, Burczyk J, Thines M. A circular chloroplast genome of *Fagus sylvatica* reveals high conservation between two individuals from Germany and one individual from Poland and an alternate direction of the small single-copy region. Forests. 2021;12: 180. doi: 10.3390/f12020180

[36] Paradis E. pegas: an R package for population genetics with an integrated-modular approach. Bioinformatics. 2010;26: 419–420. doi: 10.1093/bioinformatics/btp696 20080509

[37] Ulaszewski B, Meger J, Mishra B, Thines M, Burczyk J. Complete chloroplast genomes of *Fagus sylvatica* L. reveal sequence conservation in the inverted repeat and the presence of allelic variation in NUPTs. Genes (Basel). 2021;12. doi: 10.3390/genes12091357 34573338 PMC8468245

[38] Worth JRP, Liu L, Wei F-J, Tomaru N. The complete chloroplast genome of *Fagus crenata* (subgenus *Fagus*) and comparison with *F*. *engleriana* (subgenus *Engleriana*). PeerJ. 2019;7: e7026. doi: 10.7717/peerj.7026 31211014 PMC6557254

[39] Emms DM, Kelly S. OrthoFinder: Phylogenetic orthology inference for comparative genomics. Genome Biol. 2019;20: 1–14. doi: 10.1186/S13059-019-1832-Y 31727128 PMC6857279

[40] Kück P, Meusemann K. FASconCAT: Convenient handling of data matrices. Mol Phylogenet Evol. 2010;56: 1115–1118. doi: 10.1016/j.ympev.2010.04.024 20416383

[41] Capella-Gutiérrez S, Silla-Martínez JM, Gabaldón T. trimAl: a tool for automated alignment trimming in large-scale phylogenetic analyses. Bioinformatics. 2009;25: 1972–1973. doi: 10.1093/bioinformatics/btp348 19505945 PMC2712344

[42] Stamatakis A. RAxML version 8: a tool for phylogenetic analysis and post-analysis of large phylogenies. Bioinformatics. 2014;30: 1312–1313. doi: 10.1093/bioinformatics/btu033 24451623 PMC3998144

[43] Yu G, Smith DK, Zhu H, Guan Y, Lam TTY. ggtree: an r package for visualization and annotation of phylogenetic trees with their covariates and other associated data. Methods Ecol Evol. 2017;8: 28–36. doi: 10.1111/2041-210X.12628

[44] Bouckaert R, Vaughan TG, Barido-Sottani J, Duchêne S, Fourment M, Gavryushkina A, et al. BEAST 2.5: An advanced software platform for Bayesian evolutionary analysis. PLoS Comput Biol. 2019;15. doi: 10.1371/journal.pcbi.1006650 30958812 PMC6472827

[45] Liu S, Lorenzen ED, Fumagalli M, Li B, Harris K, Xiong Z, et al. Population genomics reveal recent speciation and rapid evolutionary adaptation in polar bears. Cell. 2014;157: 785–794. doi: 10.1016/j.cell.2014.03.054 24813606 PMC4089990

[46] Zhou BF, Yuan S, Crowl AA, Liang YY, Shi Y, Chen XY, et al. Phylogenomic analyses highlight innovation and introgression in the continental radiations of Fagaceae across the Northern Hemisphere. Nat Commun 2022 131. 2022;13: 1–14. doi: 10.1038/s41467-022-28917-1 35288565 PMC8921187

[47] Grímsson F, Grimm GW, Zetter R, Denk T. Cretaceous and Paleogene Fagaceae from North America and Greenland: evidence for a Late Cretaceous split between *Fagus* and the remaining Fagaceae. Acta Palaeobot. 2016;56: 247–305. doi: 10.1515/ACPA-2016-0016

[48] Wilf P, Nixon KC, Gandolfo MA, Cúneo NR. Eocene Fagaceae from Patagonia and Gondwanan legacy in Asian rainforests. Science. 2019;364. doi: 10.1126/SCIENCE.AAW5139 31171664

[49] Denk T, Hill RS, Simeone MC, Cannon C, Dettmann ME, Manos PS. Comment on “Eocene Fagaceae from Patagonia and Gondwanan legacy in Asian rainforests.” Science. 2019;366. doi: 10.1126/SCIENCE.AAZ2189 31727801

[50] Ritchie AM, Lo N, Ho SYW. The impact of the tree prior on molecular dating of data sets containing a mixture of inter- and intraspecies sampling. Syst Biol. 2017;66: 413–425. doi: 10.1093/sysbio/syw095 27798404

[51] Rambaut A, Drummond AJ, Xie D, Baele G, Suchard MA. Posterior summarization in Bayesian phylogenetics using Tracer 1.7. Syst Biol. 2018;67: 901–904. doi: 10.1093/sysbio/syy032 29718447 PMC6101584

[52] Puttick MN. MCMCtreeR: functions to prepare MCMCtree analyses and visualize posterior ages on trees. Bioinformatics. 2019;35: 5321–5322. doi: 10.1093/bioinformatics/btz554 31292621

[53] Inkscape Project. Inkscape. Available: https://inkscape.org

[54] Andrews S. FastQC: A quality control tool for high throughput sequence data. 2010. Available: www.bioinformatics.babraham.ac.uk/projects/fastqc

[55] Chen S, Zhou Y, Chen Y, Gu J. fastp: an ultra-fast all-in-one FASTQ preprocessor. Bioinformatics. 2018;34: i884–i890. doi: 10.1093/bioinformatics/bty560 30423086 PMC6129281

[56] Li H, Durbin R. Fast and accurate short read alignment with Burrows-Wheeler transform. Bioinformatics. 2009;25: 1754–1760. doi: 10.1093/bioinformatics/btp324 19451168 PMC2705234

[57] Li H, Handsaker B, Wysoker A, Fennell T, Ruan J, Homer N, et al. The Sequence Alignment/Map format and SAMtools. Bioinformatics. 2009;25: 2078–2079. doi: 10.1093/bioinformatics/btp352 19505943 PMC2723002

[58] Institute Broad. Picard Toolkit. 2022.

[59] McKenna A, Hanna M, Banks E, Sivachenko A, Cibulskis K, Kernytsky A, et al. The Genome Analysis Toolkit: A MapReduce framework for analyzing next-generation DNA sequencing data. Genome Res. 2010;20: 1297–1303. doi: 10.1101/gr.107524.110 20644199 PMC2928508

[60] Poplin R, Ruano-Rubio V, Depristo MA, Fennell TJ, Carneiro MO, Van Der Auwera GA, et al. Scaling accurate genetic variant discovery to tens of thousands of samples. BioRxiv 2017. doi: 10.1101/201178

[61] Danecek P, Auton A, Abecasis G, Albers CA, Banks E, DePristo MA, et al. The variant call format and VCFtools. Bioinformatics. 2011;27: 2156–2158. doi: 10.1093/bioinformatics/btr330 21653522 PMC3137218

[62] Cingolani P, Platts A, Wang LL, Coon M, Nguyen T, Wang L, et al. A program for annotating and predicting the effects of single nucleotide polymorphisms, SnpEff. Fly (Austin). 2012;6: 80–92. doi: 10.4161/fly.19695 22728672 PMC3679285

[63] Purcell S, Neale B, Todd-Brown K, Thomas L, Ferreira MAR, Bender D, et al. PLINK: a tool set for whole-genome association and population-based linkage analyses. Am J Hum Genet. 2007;81: 559–575. doi: 10.1086/519795 17701901 PMC1950838

[64] Chang CC, Chow CC, Tellier LC, Vattikuti S, Purcell SM, Lee JJ. Second-generation PLINK: rising to the challenge of larger and richer datasets. Gigascience. 2015;4: 7. doi: 10.1186/s13742-015-0047-8 25722852 PMC4342193

[65] Korneliussen TS, Albrechtsen A, Nielsen R. ANGSD: Analysis of Next Generation Sequencing Data. BMC Bioinformatics. 2014;15: 356. doi: 10.1186/s12859-014-0356-4 25420514 PMC4248462

[66] Schmidt TL, Jasper M-E, Weeks AR, Hoffmann AA. Unbiased population heterozygosity estimates from genome-wide sequence data. Methods Ecol Evol. 2021;12: 1888–1898. doi: 10.1111/2041-210X.13659

[67] Korunes KL, Samuk K. PIXY: unbiased estimation of nucleotide diversity and divergence in the presence of missing data. Mol Ecol Resour. 2021;21: 1359–1368. doi: 10.1111/1755-0998.13326 33453139 PMC8044049

[68] Lucek K, Willi Y. Drivers of linkage disequilibrium across a species’ geographic range. PLOS Genet. 2021;17: e1009477. doi: 10.1371/journal.pgen.1009477 33770075 PMC8026057

[69] Khan MA, Korban SS. Association mapping in forest trees and fruit crops. J Exp Bot. 2012;63: 4045–4060. doi: 10.1093/jxb/ers105 22511806

[70] Zhang C, Dong S-S, Xu J-Y, He W-M, Yang T-L. PopLDdecay: a fast and effective tool for linkage disequilibrium decay analysis based on variant call format files. Bioinformatics. 2019;35: 1786–1788. doi: 10.1093/bioinformatics/bty875 30321304

[71] Wood SN. Generalized additive models: An introduction with R, second edition. Gen Addit Model An Introd with R, Second Ed. 2017; 1–476. doi: 10.1201/9781315370279/GENERALIZED-ADDITIVE-MODELS-SIMON-WOOD

[72] Gibson J, Morton NE, Collins A. Extended tracts of homozygosity in outbred human populations. Hum Mol Genet. 2006;15: 789–795. doi: 10.1093/hmg/ddi493 16436455

[73] Ceballos FC, Joshi PK, Clark DW, Ramsay M, Wilson JF. Runs of homozygosity: windows into population history and trait architecture. Nat Rev Genet. 2018;19: 220–234. doi: 10.1038/nrg.2017.109 29335644

[74] Biscarini F, Cozzi P, Gaspa G, Marras G. detectRUNS: Detect runs of homozygosity and runs of heterozygosity in diploid genomes. 2019. Available: https://github.com/bioinformatics-ptp/detectRUNS

[75] Kamvar ZN, Brooks JC, Grünwald NJ. Novel R tools for analysis of genome-wide population genetic data with emphasis on clonality. Front Genet. 2015;6. doi: 10.3389/fgene.2015.00208 26113860 PMC4462096

[76] Hamming RW. Error detecting and error correcting codes. Bell Syst Tech J. 1950;29: 147–160. doi: 10.1002/J.1538-7305.1950.TB00463.X

[77] Wickham H, Averick M, Bryan J, Chang W, McGowan L, François R, et al. Welcome to the Tidyverse. J Open Source Softw. 2019;4: 1686. doi: 10.21105/joss.01686

[78] Nei M, Chesser RK. Estimation of fixation indices and gene diversities. Ann Hum Genet. 1983;47: 253–259. doi: 10.1111/j.1469-1809.1983.tb00993.x 6614868

[79] Jost L. GST and its relatives do not measure differentiation. Mol Ecol. 2008;17: 4015–4026. doi: 10.1111/j.1365-294x.2008.03887.x 19238703

[80] Knaus BJ, Grünwald NJ. VCFR: a package to manipulate and visualize variant call format data in R. Mol Ecol Resour. 2017;17: 44–53. doi: 10.1111/1755-0998.12549 27401132

[81] Privé F, Luu K, Blum MGB, McGrath JJ, Vilhjálmsson BJ. Efficient toolkit implementing best practices for principal component analysis of population genetic data. Bioinformatics. 2020;36: 4449–4457. doi: 10.1093/bioinformatics/btaa520 32415959 PMC7750941

[82] Ma S, Shi G. On rare variants in principal component analysis of population stratification. BMC Genet. 2020;21: 34. doi: 10.1186/s12863-020-0833-x 32183706 PMC7077175

[83] Wang J. Marker-based estimates of relatedness and inbreeding coefficients: An assessment of current methods. J Evol Biol. 2014;27: 518–530. doi: 10.1111/jeb.12315 24444019

[84] Jump AS, Marchant R, Peñuelas J. Environmental change and the option value of genetic diversity. Trends Plant Sci. 2009;14: 51–58. doi: 10.1016/j.tplants.2008.10.002 19042147

[85] Lalagüe H, Csilléry K, Oddou-Muratorio S, Safrana J, de Quattro C, Fady B, et al. Nucleotide diversity and linkage disequilibrium at 58 stress response and phenology candidate genes in a European beech (*Fagus sylvatica* L.) population from southeastern France. Tree Genet Genomes. 2014;10: 15–26. doi: 10.1007/S11295-013-0658-0

[86] Seifert S, Vornam B, Finkeldey R. A set of 17 single nucleotide polymorphism (SNP) markers for European beech (*Fagus sylvatica* L.). Conserv Genet Resour. 2012;4: 1045–1047. doi: 10.1007/S12686-012-9703-9

[87] Ingvarsson PK. Nucleotide polymorphism and linkage disequilibrium within and among natural populations of European aspen (*Populus tremula* L., Salicaceae). Genetics. 2005;169: 945–953. doi: 10.1534/GENETICS.104.034959 15489521 PMC1449109

[88] Butler JB, Freeman JS, Potts BM, Vaillancourt RE, Kahrood H V., Ades PK, et al. Patterns of genomic diversity and linkage disequilibrium across the disjunct range of the Australian forest tree *Eucalyptus globulus*. Tree Genet Genomes 2022 183. 2022;18: 1–18. doi: 10.1007/S11295-022-01558-7

[89] Ulaszewski B, Meger J, Burczyk J. Comparative analysis of SNP discovery and genotyping in *Fagus sylvatica* L. and *Quercus robur* L. using RADseq, GBS, and ddRAD methods. Forests. 2021;12: 1–17. doi: 10.3390/f12020222

[90] Shirasawa K, Hirakawa H, Isobe S. Analytical workflow of double-digest restriction site-associated DNA sequencing based on empirical and in silico optimization in tomato. DNA Res. 2016;23: 145–153. doi: 10.1093/dnares/dsw004 26932983 PMC4833422

[91] Payen C, Sunshine AB, Ong GT, Pogachar JL, Zhao W, Dunham MJ. High-throughput identification of adaptive mutations in experimentally evolved yeast populations. PLOS Genet. 2016;12: e1006339. doi: 10.1371/journal.pgen.1006339 27727276 PMC5065121

[92] Collevatti RG, Novaes E, Silva-Junior OB, Vieira LD, Lima-Ribeiro MS, Grattapaglia D. A genome-wide scan shows evidence for local adaptation in a widespread keystone Neotropical forest tree. Hered 2019 1232. 2019;123: 117–137. doi: 10.1038/s41437-019-0188-0 30755734 PMC6781148

[93] Ji F, Ma Q, Zhang W, Liu J, Feng Y, Zhao P, et al. A genome variation map provides insights into the genetics of walnut adaptation and agronomic traits. Genome Biol. 2021;22: 1–22. doi: 10.1186/S13059-021-02517-6 34706738 PMC8554829

[94] Kozik A, Rowan BA, Lavelle D, Berke L, Eric Schranz M, Michelmore RW, et al. The alternative reality of plant mitochondrial DNA: one ring does not rule them all. PLOS Genet. 2019;15: e1008373. doi: 10.1371/journal.pgen.1008373 31469821 PMC6742443

[95] Renner SS, Grimm GW, Kapli P, Denk T. Species relationships and divergence times in beeches: new insights from the inclusion of 53 young and old fossils in a birth–death clock model. Philos Trans R Soc B Biol Sci. 2016;371. doi: 10.1098/RSTB.2015.0135 27325832 PMC4920336

[96] Jiang L, Bao Q, He W, Fan DM, Cheng SM, López-Pujol J, et al. Phylogeny and biogeography of *Fagus* (Fagaceae) based on 28 nuclear single/low-copy loci. J Syst Evol. 2022;60: 759–772. doi: 10.1111/JSE.12695

[97] Liang D, Wang H, Zhang J, Zhao Y, Wu F. Complete chloroplast genome sequence of *Fagus longipetiolata* Seemen (Fagaceae): genome structure, adaptive evolution, and phylogenetic relationships. Life. 2022;12. doi: 10.3390/LIFE12010092 35054485 PMC8778281

[98] Ivy-Ochs S, Kerschner H, Reuther A, Preusser F, Heine K, Maisch M, et al. Chronology of the last glacial cycle in the European Alps. J Quat Sci. 2008;23: 559–573. doi: 10.1002/JQS.1202

[99] Liepelt S, Cheddadi R, de Beaulieu JL, Fady B, Gömöry D, Hussendörfer E, et al. Postglacial range expansion and its genetic imprints in *Abies alba* (Mill.)—A synthesis from palaeobotanic and genetic data. Rev Palaeobot Palynol. 2009;153: 139–149. doi: 10.1016/J.REVPALBO.2008.07.007

[100] Piotti A, Leonarduzzi C, Postolache D, Bagnoli F, Spanu I, Brousseau L, et al. Unexpected scenarios from Mediterranean refugial areas: disentangling complex demographic dynamics along the Apennine distribution of silver fir. J Biogeogr. 2017;44: 1547–1558. doi: 10.1111/JBI.13011

[101] Torre S, Sebastiani F, Burbui G, Pecori F, Pepori AL, Passeri I, et al. Novel insights into refugia at the southern margin of the distribution range of the endangered species *Ulmus laevis*. Front Plant Sci. 2022;13: 145. doi: 10.3389/FPLS.2022.826158/BIBTEX PMC8886209 35242155

[102] Schmitt T, Fritz U, Delfino M, Ulrich W, Habel JC. Biogeography of Italy revisited: genetic lineages confirm major phylogeographic patterns and a pre-Pleistocene origin of its biota. Front Zool. 2021;18: 1–13. doi: 10.1186/S12983-021-00418-9 34187502 PMC8240252

[103] Baumbach L, Niamir A, Hickler T, Yousefpour R. Regional adaptation of European beech (*Fagus sylvatica*) to drought in Central European conditions considering environmental suitability and economic implications. Reg Environ Chang. 2019;19: 1159–1174. doi: 10.1007/s10113-019-01472-0

[104] Tegel W, Seim A, Hakelberg D, Hoffmann S, Panev M, Westphal T, et al. A recent growth increase of European beech (*Fagus sylvatica* L.) at its Mediterranean distribution limit contradicts drought stress. Eur J For Res. 2014;133: 61–71. doi: 10.1007/s10342-013-0737-7

[105] Roibu CC, Palaghianu C, Nagavciuc V, Ionita M, Sfecla V, Mursa A, et al. The response of beech (*Fagus sylvatica* L.) populations to climate in the easternmost sites of its European distribution. Plants. 2022;11. doi: 10.3390/plants11233310 36501348 PMC9738208

