# Supplementary material for: First genome-wide data from Italian European beech (Fagus sylvatica L.): Strong and ancient differentiation between Alps and Apennines
Source: PLoS One. 2023 Jul 20;18(7):e0288986. doi: 10.1371/journal.pone.0288986 (PMC10358878; doi:10.1371/journal.pone.0288986)
Supplement: S1 Table — For each sample, the average depth of coverage, No. of SNPs and SNP density are reported (inferred from the filtered VCF files), together with individual-based estimates of genome-wide diversity (F = inbreeding coefficient computed using PLINK; O(Hom) = Observed number of homozygous genotypes; E(Hom) = Expected number of homozygous genotypes). (DOCX) [file pone.0288986.s001.docx]

|  |  | **Depth of coverage** | | | **SNPs polymorphism** | | | **Individual-based diversity** | | | |
| --- | --- | --- | --- | --- | --- | --- | --- | --- | --- | --- | --- |
| **Sample** | **Origin** | **Avg** | **St. Dev.** | **N° SNPs** | | **Avg SNPs**  **density** (SNP/kb) | **F** | | **O(Hom)** | **E(Hom)** | **GW-Het** |
| ALP01 | Val di Cembra (Italy; Eastern Alps) | 9.2960 | 56.0696 | 3083784 | | 5.7598 | 0.5525 | | 629602 | 513100 | 0.0159 |
| ALP02 | Val di Cembra (Italy; Eastern Alps) | 18.7440 | 60.5657 | 3837319 | | 7.1672 | 0.7137 | | 567603 | 440800 | 0.0168 |
| ALP03 | Val di Cembra (Italy; Eastern Alps) | 18.5424 | 72.4926 | 3848357 | | 7.1878 | 0.5447 | | 631552 | 515900 | 0.0177 |
| ALP04 | Val di Cembra (Italy; Eastern Alps) | 17.0734 | 71.6467 | 3617697 | | 6.7570 | 0.5633 | | 637545 | 517400 | 0.0166 |
| APE01 | Maresca (Italy; North Appennines) | 15.2042 | 65.3209 | 3604323 | | 6.7320 | 0.6291 | | 591503 | 471000 | 0.0174 |
| APE02 | Maresca (Italy; North Appennines) | 17.9685 | 66.4514 | 3763102 | | 7.0286 | 0.5848 | | 621956 | 501300 | 0.0180 |
| APE03 | Maresca (Italy; North Appennines) | 17.0430 | 68.4087 | 3730918 | | 6.9684 | 0.6005 | | 616006 | 494900 | 0.0179 |
| APE04 | Maresca (Italy; North Appennines) | 16.4861 | 55.4527 | 3766085 | | 7.0341 | 0.6014 | | 615428 | 494500 | 0.0179 |
| APE05 | Maresca (Italy; North Appennines) | 16.5049 | 57.0912 | 3793367 | | 7.0851 | 0.5831 | | 609903 | 492500 | 0.0182 |
| BHAGA^1^ | Kellerwald-Edersee National Park (Germany) | 16.8499 | 91.0445 | 1870596 | | 3.4938 | 0.7605 | | 952654 | 709300 | / |
| JAMY^1^ | Jamy Nature Reserve (Poland) | 14.6146 | 45.768 | 3456790 | | 6.4564 | 0.6217 | | 617327 | 491800 | / |

**S1 Table. List of the analyzed European beech nuclear genomes.** For each sample, the average depth of coverage, No. of SNPs and SNP density are reported (inferred from the filtered VCF files), together with individual-based estimates of genome-wide diversity (F = inbreeding coefficient computed using PLINK; O(Hom) = Observed number of homozygous genotypes; E(Hom) = Expected number of homozygous genotypes); GW-Het = genome-wide heterozygosity of the samples sequenced in the present study, computed with ANGSD.

^1^ for BHAGA and JAMY (available reads downloaded from GenBank), all the reported parameters were computed after downsampling of the original reads dataset (see Materials and Methods)
