# Supplementary material for: First genome-wide data from Italian European beech (Fagus sylvatica L.): Strong and ancient differentiation between Alps and Apennines
Source: PLoS One. 2023 Jul 20;18(7):e0288986. doi: 10.1371/journal.pone.0288986 (PMC10358878; doi:10.1371/journal.pone.0288986)
Supplement: S2 Table — The annotated proteomes correspondent to each accession were downloaded from the NCBI website. (DOCX) [file pone.0288986.s002.docx]

**S2 Table. List of all Fagaceae chloroplast and mitochondrial genomes used for phylogenetic inference.** The annotated proteomes correspondent to each accession were downloaded from the NCBI website.

| **Accession** | **Species** | **Type** |
| --- | --- | --- |
| MT627605.1 | *Castanopsis sclerophylla* | cpDNA |
| MZ433364.1 | *Castanopsis hystrix* | cpDNA |
| MZ562567.1 | *Fagus longipetiolata* | cpDNA |
| NC_014674.1 | *Castanea mollissima* | cpDNA |
| NC_029490.1 | *Quercus baronii* | cpDNA |
| NC_031356.1 | *Quercus variabilis* | cpDNA |
| NC_031357.1 | *Quercus dolicholepis* | cpDNA |
| NC_033881.1 | *Castanea henryi* | cpDNA |
| NC_036370.1 | *Quercus tarokoensis* | cpDNA |
| NC_036929.1 | *Fagus engleriana* | cpDNA |
| NC_039428.1 | *Quercus chenii* | cpDNA |
| NC_039429.1 | *Quercus acutissima* | cpDNA |
| NC_039749.1 | *Castanea seguinii* | cpDNA |
| NC_041252.1 | *Fagus crenata* | cpDNA |
| NC_041437.1 | *Fagus sylvatica* | cpDNA |
| NC_046583.1 | *Quercus bawanglingensis* | cpDNA |
| NC_048488.1 | *Quercus phillyraeoides* | cpDNA |
| NC_053352.1 | *Fagus japonica* | cpDNA |
| NC_057119.1 | *Castanopsis carlesii* | cpDNA |
| MN199236.1 | *Quercus variabilis* | mtDNA |
| MW582695.1 | *Fagus sylvatica* | mtDNA |
| MW771358.1 | *Fagus sylvatica* | mtDNA |
| NC_050960.1 | *Fagus sylvatica* | mtDNA |
| OW028777.1 | *Quercus robur* | mtDNA |
