# Supplementary material for: First genome-wide data from Italian European beech (Fagus sylvatica L.): Strong and ancient differentiation between Alps and Apennines
Source: PLoS One. 2023 Jul 20;18(7):e0288986. doi: 10.1371/journal.pone.0288986 (PMC10358878; doi:10.1371/journal.pone.0288986)
Supplement: S3 Table — Summary of SnpEff annotation with the number of SNPs found in each effect type category. (DOCX) [file pone.0288986.s003.docx]

**S3 Table. Functional annotations of the potential SNP effects.** Summary of SnpEff annotation with the number of SNPs found in each effect type category.

| **Type (alphabetical order)** | **Count** | **Percent** |
| --- | --- | --- |
| **3_prime_UTR_variant** | 2438 | 0,87% |
| **5_prime_UTR_premature_start_codon_gain_variant** | 3345 | 0,12% |
| **5_prime_UTR_variant** | 20503 | 0,73% |
| **downstream_gene_variant** | 733515 | 26,26% |
| **initiator_codon_variant** | 35 | 0.001% |
| **intergenic_region** | 778498 | 27,87% |
| **intron_variant** | 230082 | 8,24% |
| **missense_variant** | 132606 | 4,75% |
| **splice_acceptor_variant** | 1065 | 0,04% |
| **splice_donor_variant** | 828 | 0,03% |
| **splice_region_variant** | 8928 | 0,32% |
| **start_lost** | 275 | 0,01% |
| **stop_gained** | 6611 | 0,24% |
| **stop_lost** | 356 | 0,01% |
| **stop_retained_variant** | 118 | 0,00% |
| **synonymous_variant** | 79743 | 2,86% |
| **upstream_gene_variant** | 772298 | 27,65% |
| **Total** | 2771244 | 100,00% |
| **Non genes** | 2284311 | 81,78% |
| **Genes** | 486933 | 18,22% |
| **Genes-excluding intron and synonymous** | 177108 | 7,13% |
