# Supplementary material for: First genome-wide data from Italian European beech (Fagus sylvatica L.): Strong and ancient differentiation between Alps and Apennines
Source: PLoS One. 2023 Jul 20;18(7):e0288986. doi: 10.1371/journal.pone.0288986 (PMC10358878; doi:10.1371/journal.pone.0288986)
Supplement: S4 Table — (DOCX) [file pone.0288986.s004.docx]

| **Pairwise comparisons: ALP** | **r** |
| --- | --- |
| ALP02-ALP01 | -0,3404 |
| ALP03-ALP01 | -0,3799 |
| ALP03-ALP02 | -0,3782 |
| ALP04-ALP01 | -0,3780 |
| ALP04-ALP02 | -0,3311 |
| ALP04-ALP03 | -0,3920 |
| **ALP average** | -0,3666 |
| **ALP s.d.** | 0,0246 |

**S4 Table. Pairwise relatedness coefficients (r) between the individuals sampled within each forest stand (ALP and APE).** Values were estimated using PLINK (–make-rel command).

| **Pairwise comparisons: APE** | **r** |
| --- | --- |
| APE02-APE01 | -0,3074 |
| APE03-APE01 | -0,3020 |
| APE03-APE02 | -0,3151 |
| APE04-APE01 | -0,2814 |
| APE04-APE02 | -0,3078 |
| APE04-APE03 | -0,2788 |
| APE05-APE01 | -0,2650 |
| APE05-APE02 | -0,3106 |
| APE05-APE03 | -0,3017 |
| APE05-APE04 | -0,3197 |
| **APE average** | -0,2989 |
| **APE s.d.** | 0,0178 |
