# Supplementary material for: First genome-wide data from Italian European beech (Fagus sylvatica L.): Strong and ancient differentiation between Alps and Apennines
Source: PLoS One. 2023 Jul 20;18(7):e0288986. doi: 10.1371/journal.pone.0288986 (PMC10358878; doi:10.1371/journal.pone.0288986)
Supplement: S5 Table — (DOCX) [file pone.0288986.s005.docx]

| **Organelle** | **Sample** | **GenBank accession number** |
| --- | --- | --- |
| Chloroplast | ALP1 | OQ428820 |
| Chloroplast | ALP2 | OQ428821 |
| Chloroplast | ALP3 | OQ428822 |
| Chloroplast | ALP4 | OQ428823 |
| Chloroplast | APE1 | OQ428824 |
| Chloroplast | APE2 | OQ428825 |
| Chloroplast | APE3 | OQ428826 |
| Chloroplast | APE4 | OQ428827 |
| Chloroplast | APE5 | OQ428828 |
| Mitochondrial | APE1 | OQ428829 |
| Mitochondrial | APE2 | OQ428830 |
| Mitochondrial | APE4 | OQ428831 |
| Mitochondrial | APE5 | OQ428832 |
| Mitochondrial | APE3 | OQ428833 |
| Mitochondrial | ALP2 | OQ428834 |
| Mitochondrial | ALP3 | OQ428835 |
| Mitochondrial | ALP4 | OQ428836 |
| Mitochondrial | ALP1 | OQ428837 |

**S5 Table. GenBank accession numbers GenBank of the sequences of the chloroplast and mitochondrial genomes produced in the present study.**
