# Supplementary figures and images for: First genome-wide data from Italian European beech (Fagus sylvatica L.): Strong and ancient differentiation between Alps and Apennines
Source: PLoS One. 2023 Jul 20;18(7):e0288986. doi: 10.1371/journal.pone.0288986 (PMC10358878; doi:10.1371/journal.pone.0288986)

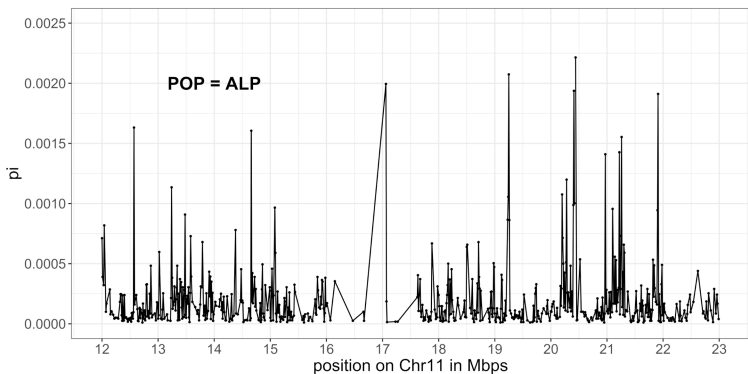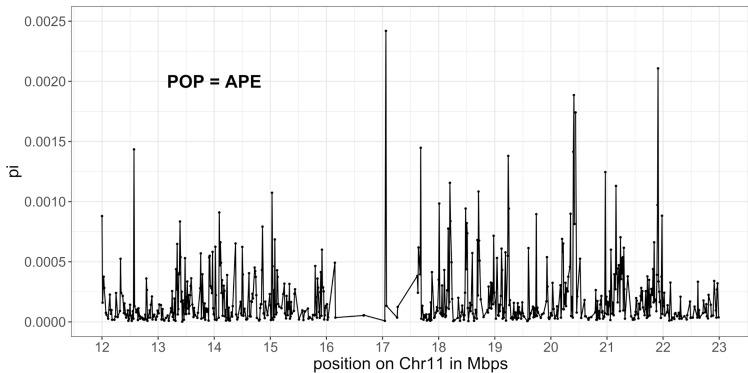

Supplement: S1 Fig — The plot shows the results of a sliding window analysis (window length = 10 kb) of nucleotide diversity per site (π) on chromosome 11, where large organellar genome insertions (from about Mb 16 to Mb 18) had been identified in a previous study [25]. (PDF) [file pone.0288986.s006.pdf]

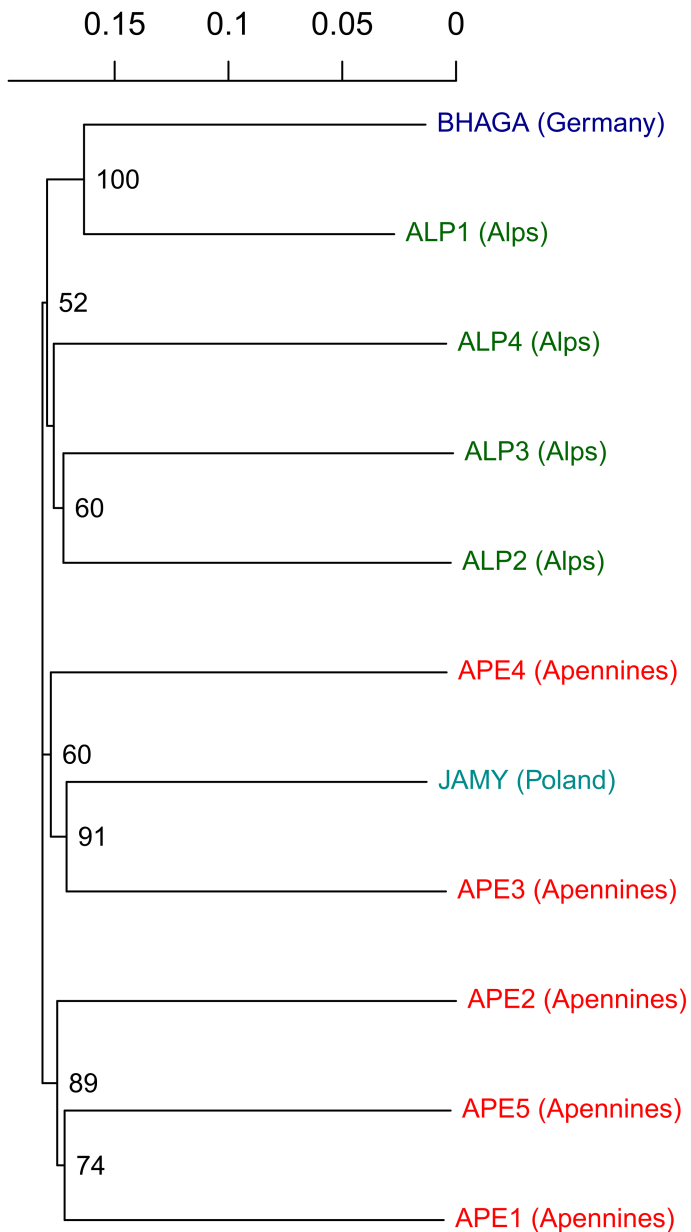

Supplement: S2 Fig — The analysis is based on pairwise identity-by-state (IBS) distance calculated from the 38,271 genome-wide SNPs dataset (after filtering for MAF ≥ 5% and LD pruning at r2 < 0.50). (PDF) [file pone.0288986.s007.pdf]

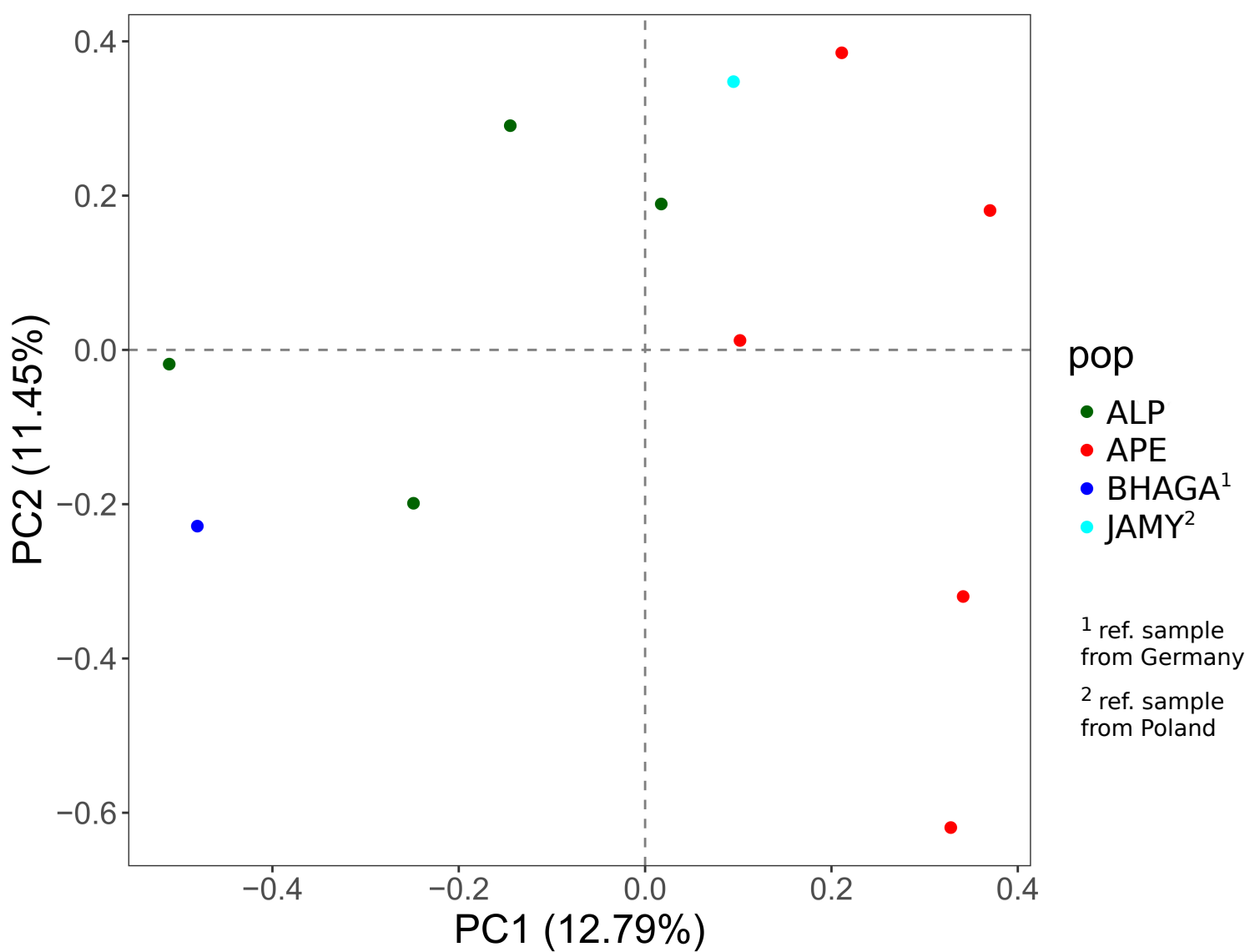

Supplement: S3 Fig — Individuals are plotted on the biplot of the first two principal components; the dataset includes nine Italian samples from the Alpine (ALP) and Apennine (APE) populations, one German (BHAGA) and one Polish sample (JAMY). (PDF) [file pone.0288986.s008.pdf]

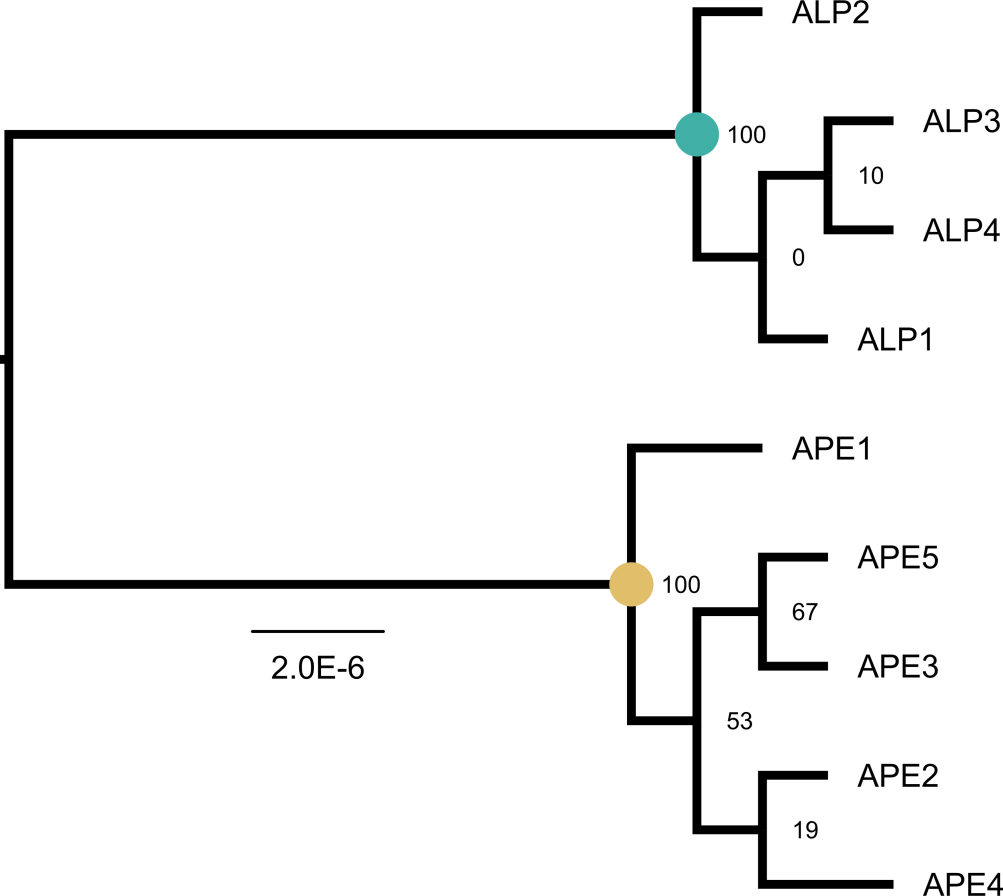

Supplement: S4 Fig — Mitochondrial data show a sharp separation between the Alpine and the Apennine populations (these two clades are indicated by dots of different colors at the node), with very high bootstrap values. The two clades cannot be further divided into subclades reliably, as shown by the very low bootstrap supports inside these two groups. The tree was midpoint rooted. A scale bar indicating the substitutions per site is indicated below the tree. (PDF) [file pone.0288986.s009.pdf]

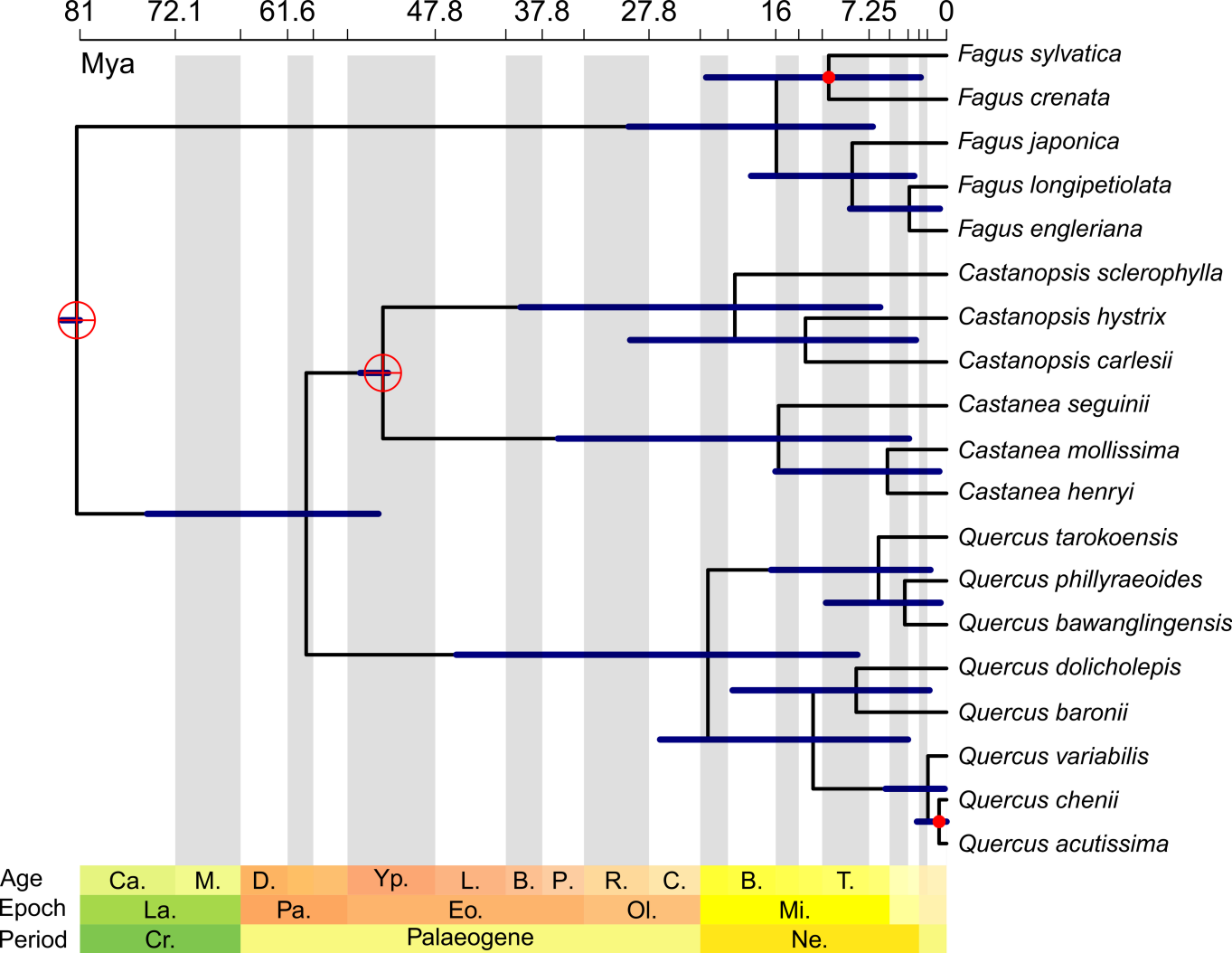

Supplement: S5 Fig — Big and crossed red circles indicate fossil calibrated nodes. Full red circles indicate nodes for which posterior probabilities were below 60. Blue bars show the 95% highest posterior densities (HPDs) of the date estimates, representing the shortest interval on the posterior density of age estimates for a 95% confidence level. (PDF) [file pone.0288986.s010.pdf]

## Number of effects by region

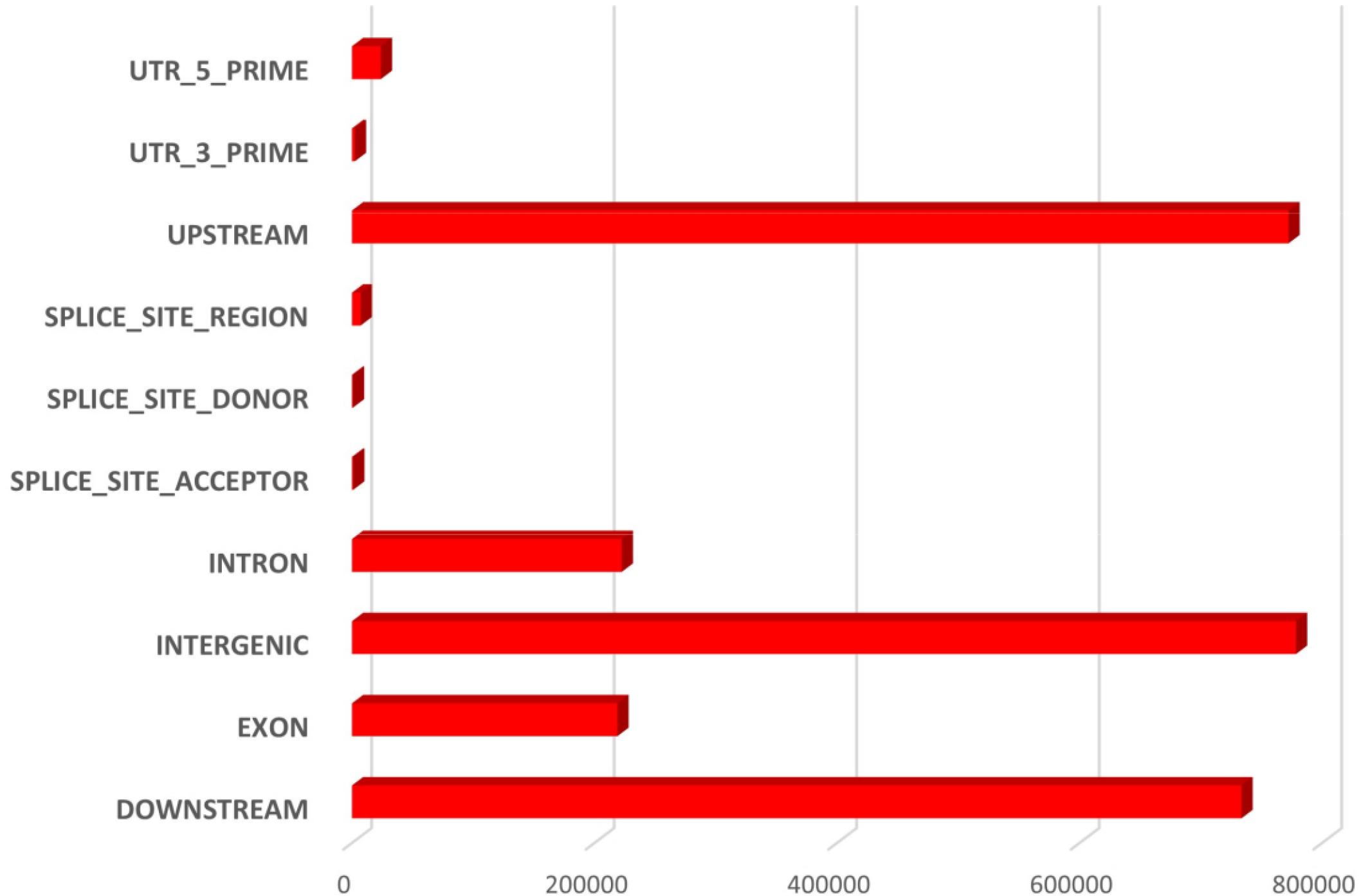

Supplement: S6 Fig — SNPs classification according to their effect and distribution across genomic regions. The X-axis displays the variants number, and the Y-axis the different regions. (PDF) [file pone.0288986.s011.pdf]
